# Limitation of life-sustaining therapies in critically ill patients with COVID-19: a descriptive epidemiological investigation from the COVID-ICU study

**DOI:** 10.1186/s13054-023-04349-1

**Published:** 2023-03-11

**Authors:** Mikhael Giabicani, Christophe Le Terrier, Antoine Poncet, Bertrand Guidet, Jean-Philippe Rigaud, Jean-Pierre Quenot, Marie-France Mamzer, Jérôme Pugin, Emmanuel Weiss, Simon Bourcier, COVID-ICU study investigators, Alain Mercat, Pierre Asfar, François Beloncle, Julien Demiselle, Tài Pham, Arthur Pavot, Xavier Monnet, Christian Richard, Alexandre Demoule, Martin Dres, Julien Mayaux, Alexandra Beurton, Cédric Daubin, Richard Descamps, Aurélie Joret, Damien Du Cheyron, Frédéric Pene, Jean-Daniel Chiche, Mathieu Jozwiak, Paul Jaubert, Guillaume Voiriot, Muriel Fartoukh, Marion Teulier, Clarisse Blayau, Erwen L’Her, Cécile Aubron, Laetitia Bodenes, Nicolas Ferriere, Johann Auchabie, Anthony Le Meur, Sylvain Pignal, Thierry Mazzoni, Jean-Pierre Quenot, Pascal Andreu, Jean-Baptiste Roudau, Marie Labruyère, Saad Nseir, Sébastien Preau, Julien Poissy, Daniel Mathieu, Sarah Benhamida, Rémi Paulet, Nicolas Roucaud, Martial Thyrault, Florence Daviet, Sami Hraiech, Gabriel Parzy, Aude Sylvestre, Sébastien Jochmans, Anne-Laure Bouilland, Mehran Monchi, Marc Danguy des Déserts, Quentin Mathais, Gwendoline Rager, Pierre Pasquier, Jean Reignier, Amélie Seguin, Charlotte Garret, Emmanuel Canet, Jean Dellamonica, Clément Saccheri, Romain Lombardi, Yanis Kouchit, Sophie Jacquier, Armelle Mathonnet, Mai-Ahn Nay, Isabelle Runge, Frédéric Martino, Laure Flurin, Amélie Rolle, Michel Carles, Rémi Coudroy, Arnaud W. Thille, Jean-Pierre Frat, Maeva Rodriguez, Pascal Beuret, Audrey Tientcheu, Arthur Vincent, Florian Michelin, Fabienne Tamion, Dorothée Carpentier, Déborah Boyer, Gaetan Beduneau, Valérie Gissot, Stéphan Ehrmann, Charlotte Salmon Gandonniere, Djlali Elaroussi, Agathe Delbove, Yannick Fedun, Julien Huntzinger, Eddy Lebas, Grâce Kisoka, Céline Grégoire, Stella Marchetta, Bernard Lambermont, Laurent Argaud, Thomas Baudry, Pierre-Jean Bertrand, Auguste Dargent, Christophe Guitton, Nicolas Chudeau, Mickaël Landais, Cédric Darreau, Alexis Ferre, Antoine Gros, Guillaume Lacave, Fabrice Bruneel, Mathilde Neuville, Jérôme Devaquet, Guillaume Tachon, Richard Gallot, Riad Chelha, Arnaud Galbois, Anne Jallot, Ludivine Chalumeau Lemoine, Khaldoun Kuteifan, Valentin Pointurier, Louise-Marie Jandeaux, Joy Mootien, Charles Damoisel, Benjamin Sztrymf, Matthieu Schmidt, Alain Combes, Juliette Chommeloux, Charles Edouard Luyt, Frédérique Schortgen, Leon Rusel, Camille Jung, Florent Gobert, Damien Vimpere, Lionel Lamhaut, Bertrand Sauneuf, Liliane Charrrier, Julien Calus, Isabelle Desmeules, Benoît Painvin, Jean-Marc Tadie, Vincent Castelain, Baptiste Michard, Jean-Etienne Herbrecht, Mathieu Baldacini, Vincent Castelain, Baptiste Michard, Jean-Etienne Herbrecht, Mathieu Baldacini, Nicolas Weiss, Sophie Demeret, Clémence Marois, Benjamin Rohaut, Pierre-Henri Moury, Anne-Charlotte Savida, Emmanuel Couadau, Mathieu Série, Nica Alexandru, Cédric Bruel, Candice Fontaine, Sonia Garrigou, Juliette Courtiade Mahler, Maxime Leclerc, Michel Ramakers, Pierre Garçon, Nicole Massou, Ly Van Vong, Juliane Sen, Nolwenn Lucas, Franck Chemouni, Annabelle Stoclin, Alexandre Avenel, Henri Faure, Angélie Gentilhomme, Sylvie Ricome, Paul Abraham, Céline Monard, Julien Textoris, Thomas Rimmele, Florent Montini, Gabriel Lejour, Thierry Lazard, Isabelle Etienney, Younes Kerroumi, Claire Dupuis, Marine Bereiziat, Elisabeth Coupez, François Thouy, Clément Hoffmann, Nicolas Donat, Anne Chrisment, Rose-Marie Blot, Antoine Kimmoun, Audrey Jacquot, Matthieu Mattei, Bruno Levy, Ramin Ravan, Loïc Dopeux, Jean-Mathias Liteaudon, Delphine Roux, Brice Rey, Radu Anghel, Deborah Schenesse, Vincent Gevrey, Jermy Castanera, Philippe Petua, Benjamin Madeux, Otto Hartman, Michael Piagnerelli, Anne Joosten, Cinderella Noel, Patrick Biston, Thibaut Noel, Gurvan L. E. Bouar, Messabi Boukhanza, Elsa Demarest, Marie-France Bajolet, Nathanaël Charrier, Audrey Quenet, Cécile Zylberfajn, Nicolas Dufour, Buno Mégarbane, Sébastian Voicu, Nicolas Deye, Isabelle Malissin, François Legay, Matthieu Debarre, Nicolas Barbarot, Pierre Fillatre, Bertrand Delord, Thomas Laterrade, Tahar Saghi, Wilfried Pujol, Pierre Julien Cungi, Pierre Esnault, Mickael Cardinale, Vivien Hong Tuan Ha, Grégory Fleury, Marie-Ange Brou, Daniel Zafimahazo, David Tran-Van, Patrick Avargues, Lisa Carenco, Nicolas Robin, Alexandre Ouali, Lucie Houdou, Christophe Le Terrier, Noémie Suh, Steve Primmaz, Jérome Pugin, Emmanuel Weiss, Tobias Gauss, Jean-Denis Moyer, Catherine Paugam Burtz, Béatrice La Combe, Rolland Smonig, Jade Violleau, Pauline Cailliez, Jonathan Chelly, Antoine Marchalot, Cécile Saladin, Christelle Bigot, Pierre-Marie Fayolle, Jules Fatséas, Amr Ibrahim, Dabor Resiere, Rabih Hage, Clémentine Cholet, Marie Cantier, Pierre Trouiler, Philippe Montravers, Brice Lortat-Jacob, Sebastien Tanaka, Alexy Tran Dinh, Jacques Duranteau, Anatole Harrois, Guillaume Dubreuil, Marie Werner, Anne Godier, Sophie Hamada, Diane Zlotnik, Hélène Nougue, Armand Mekontso-Dessap, Guillaume Carteaux, Keyvan Razazi, Nicolas De Prost, Nicolas Mongardon, Meriam Lamraoui, Claire Alessandri, Quentin de Roux, Charles de Roquetaillade, Benjamin G. Chousterman, Alexandre Mebazaa, Etienne Gayat, Marc Garnier, Emmanuel Pardo, Lea Satre-Buisson, Christophe Gutton, Elise Yvin, Clémence Marcault, Elie Azoulay, Michael Darmon, Hafid Ait Oufella, Geoffroy Hariri, Tomas Urbina, Sandie Mazerand, Nicholas Heming, Francesca Santi, Pierre Moine, Djillali Annane, Adrien Bouglé, Edris Omar, Aymeric Lancelot, Emmanuelle Begot, Gaétan Plantefeve, Damien Contou, Hervé Mentec, Olivier Pajot, Stanislas Faguer, Olivier Cointault, Laurence Lavayssiere, Marie-Béatrice Nogier, Matthieu Jamme, Claire Pichereau, Jan Hayon, Hervé Outin, François Dépret, Maxime Coutrot, Maité Chaussard, Lucie Guillemet, Pierre Goffin, Romain Thouny, Julien Guntz, Laurent Jadot, Romain Persichini, Vanessa Jean-Michel, Hugues Georges, Thomas Caulier, Gaël Pradel, Marie-Hélène Hausermann, Thi My Hue Nguyen-Valat, Michel Boudinaud, Emmanuel Vivier, Sylvène Rosseli, Gaël Bourdin, Christian Pommier, Marc Vinclair, Simon Poignant, Sandrine Mons, Wulfran Bougouin, Franklin Bruna, Quentin Maestraggi, Christian Roth, Laurent Bitker, François Dhelft, Justine Bonnet-Chateau, Mathilde Filippelli, Tristan Morichau-Beauchant, Stéphane Thierry, Charlotte Le Roy, Mélanie Saint Jouan, Bruno Goncalves, Aurélien Mazeraud, Matthieu Daniel, Tarek Sharshar, Cyril Cadoz, Rostane Gaci, Sébastien Gette, Guillaune Louis, Sophe-Caroline Sacleux, Marie-Amélie Ordan, Aurélie Cravoisy, Marie Conrad, Guilhem Courte, Sébastien Gibot, Younès Benzidi, Claudia Casella, Laurent Serpin, Jean-Lou Setti, Marie-Catherine Besse, Anna Bourreau, Jérôme Pillot, Caroline Rivera, Camille Vinclair, Marie-Aline Robaux, Chloé Achino, Marie-Charlotte Delignette, Tessa Mazard, Frédéric Aubrun, Bruno Bouchet, Aurélien Frérou, Laura Muller, Charlotte Quentin, Samuel Degoul, Xavier Stihle, Claude Sumian, Nicoletta Bergero, Bernard Lanaspre, Hervé Quintard, Eve Marie Maiziere, Pierre-Yves Egreteau, Guillaume Leloup, Florin Berteau, Marjolaine Cottrel, Marie Bouteloup, Matthieu Jeannot, Quentin Blanc, Julien Saison, Isabelle Geneau, Romaric Grenot, Abdel Ouchike, Pascal Hazera, Anne-Lyse Masse, Suela Demiri, Corinne Vezinet, Elodie Baron, Deborah Benchetrit, Antoine Monsel, Grégoire Trebbia, Emmanuelle Schaack, Raphaël Lepecq, Mathieu Bobet, Christophe Vinsonneau, Thibault Dekeyser, Quentin Delforge, Imen Rahmani, Bérengère Vivet, Jonathan Paillot, Lucie Hierle, Claire Chaignat, Sarah Valette, Benoït Her, Jennifier Brunet, Mathieu Page, Fabienne Boiste, Anthony Collin, Florent Bavozet, Aude Garin, Mohamed Dlala, Kais Mhamdi, Bassem Beilouny, Alexandra Lavalard, Severine Perez, Benoit Veber, Pierre-Gildas Guitard, Philippe Gouin, Anna Lamacz, Fabienne Plouvier, Bertrand P. Delaborde, Aïssa Kherchache, Amina Chaalal, Jean-Damien Ricard, Marc Amouretti, Santiago Freita-Ramos, Damien Roux, Jean-Michel Constantin, Mona Assefi, Marine Lecore, Agathe Selves, Florian Prevost, Christian Lamer, Ruiying Shi, Lyes Knani, Sébastien Pili Floury, Lucie Vettoretti, Michael Levy, Lucile Marsac, Stéphane Dauger, Sophie Guilmin-Crépon, Hadrien Winiszewski, Gael Piton, Thibaud Soumagne, Gilles Capellier, Jean-Baptiste Putegnat, Frédérique Bayle, Maya Perrou, Ghyslaine Thao, Guillaume Géri, Cyril Charron, Xavier Repessé, Antoine Vieillard-Baron, Mathieu Guilbart, Pierre-Alexandre Roger, Sébastien Hinard, Pierre-Yves Macq, Kevin Chaulier, Sylvie Goutte, Patrick Chillet, Anaïs Pitta, Barbara Darjent, Amandine Bruneau, Sigismond Lasocki, Maxime Leger, Soizic Gergaud, Pierre Lemarie, Nicolas Terzi, Carole Schwebel, Anaïs Dartevel, Louis-Marie Galerneau, Jean-Luc Diehl, Caroline Hauw-Berlemont, Nicolas Péron, Emmanuel Guérot, Abolfazl Mohebbi Amoli, Michel Benhamou, Jean-Pierre Deyme, Olivier Andremont, Diane Lena, Julien Cady, Arnaud Causeret, Arnaud De La Chapelle, Christophe Cracco, Stéphane Rouleau, David Schnell, Camille Foucault, Cécile Lory, Thibault Chapelle, Vincent Bruckert, Julie Garcia, Abdlazize Sahraoui, Nathalie Abbosh, Caroline Bornstain, Pierre Pernet, Florent Poirson, Ahmed Pasem, Philippe Karoubi, Virginie Poupinel, Caroline Gauthier, François Bouniol, Philippe Feuchere, Florent Bavozet, Anne Heron, Serge Carreira, Malo Emery, Anne Sophie Le Floch, Luana Giovannangeli, Nicolas Herzog, Christophe Giacardi, Thibaut Baudic, Chloé Thill, Said Lebbah, Jessica Palmyre, Florence Tubach, David Hajage, Nicolas Bonnet, Nathan Ebstein, Stéphane Gaudry, Yves Cohen, Julie Noublanche, Olivier Lesieur, Arnaud Sément, Isabel Roca-Cerezo, Michel Pascal, Nesrine Sma, Gwenhaël Colin, Jean-Claude Lacherade, Gauthier Bionz, Natacha Maquigneau, Pierre Bouzat, Michel Durand, Marie-Christine Hérault, Jean-Francois Payen

**Affiliations:** 1 Department of Anaesthesiology and Critical Care, Beaujon Hospital, DMU Parabol, AP-HP Nord, Paris, France; 2 Centre de Recherche des Cordeliers, Université Paris Cité, Inserm, Laboratoire ETREs, Sorbonne Université, Paris, France; 3 Division of Intensive Care, Geneva University Hospitals, Faculty of Medicine, University of Geneva, 4 Rue Gabrielle-Perret-Gentil, 1211 Geneva 14, Switzerland; 4 Clinical Research Centre, Faculty of Medicine, University of Geneva, Geneva, Switzerland; 5 Division of Clinical Epidemiology, Department of Health and Community Medicine, University Hospitals of Geneva, Geneva, Switzerland; 6 Service de Réanimation Médicale, Assistance Publique-Hôpitaux de Paris, Hôpital Saint-Antoine, Paris, France; 7 Réanimation Polyvalente, Centre Hospitalier de Dieppe, Dieppe, France; 8 Department of Intensive Care, François Mitterrand University Hospital, Dijon, France; 9 Unité Fonctionnelle d’Ethique Médicale, Hôpital Necker-Enfants Malades, APHP, Paris, France; 10 CHU Angers, Angers, France; 11 APHP - Hôpital Bicêtre, Le Kremlin-Bicêtre, France; 12 APHP - Hôpital Pitié Salpêtrière, Paris, France; 13 CHU Caen Normandie - Hôpital Côte de Nacre, Caen, France; 14 APHP - Hôpital Cochin, Paris, France; 15 APHP - Hôpital Tenon, Paris, France; 16 CHRU de Brest – La Cavale Blanche, Brest, France; 17 Centre Hospitalier de Cholet, Cholet, France; 18 CHU Dijon Bourgogne, Dijon, France; 19 CHU Lille - Hôpital Roger Salengero, Lille, France; 20 Groupe Hospitalier Nord Essonne, Longjumeau, France; 21 APHM - Hopital Nord, Marseille, France; 22 Hôpital de Melun-Sénart, Melun, France; 23 Élément Militaire de Réanimation du SSA, Mulhouse, France; 24 CHU Nantes - Hôpital Hotel Dieu, Nantes, France; 25 CHU Nice - Hôpital Archet, Nice, France; 26 Centre Hospitalier d’Orléans, Orléans, France; 27 Centre Hospitalier Universitaire de La Guadeloupe, Pointe-a-Pitre, France; 28 Hôpital de La Milétrie, Poitiers, France; 29 Centre Hospitalier Roanne, Roanne, France; 30 CHU Rouen - Hôpital Charles Nicolle, Rouen, France; 31 CHRU Tours - Hôpital Bretonneau, Tours, France; 32 Centre Hospitalier Bretagne Atlantique, Vannes, France; 33 CHU Liège, Liège, Belgium; 34 Hospices Civils de Lyon - Hôpital Edouard Herriot, Lyon, France; 35 Centre Hospitalier du Mans, Le Mans, France; 36 Centre Hospitalier de Versailles, Le Chesnay, France; 37 Hôpital Foch, Suresnes, France; 38 Hôpital Claude Galien, Quincy Sous Senart, France; 39 GHR Mulhouse Sud-Alsace, Mulhouse, France; 40 APHP – Hôpital Antoine Béclère, Clamart, France; 41 APHP - Hôpital Pitié-Salpêtrière, Paris, France; 42 Hôpital Intercommunal de Créteil, Créteil, France; 43 Hospices Civils de Lyon - Hôpital Neurologique, Lyon, France; 44 APHP – Hôpital Necker, Paris, France; 45 Centre Hospitalier Public du Cotentin - Hôpital Pasteur, Cherbourg-en-Cotentin, France; 46 CHU Rennes - Hôpital du Pontchaillou, Rennes, France; 47 CHU Strasbourg - Hôpital Hautepierre, Strasbourg, France; 48 Centre Hospitalier Territorial Gaston-Bourret, Nouméa, France; 49 Centre Hospitalier Compiègne-Noyon, Compiègne, France; 50 Groupe Hospitalier Saint-Joseph, Paris, France; 51 Centre Hospitalier Mémorial de Saint-Lô, Saint-Lô, France; 52 Grand Hôpital de l’Est Francilien, Jossigny, France; 53 Gustave Roussy, Villejuif, France; 54 Centre Hospitalier Intercommunal Robert Ballanger, Aulnay-Sous-Bois, France; 55 Hospices Civiles de Lyon - Hôpital Edouard Herriot, Lyon, France; 56 Centre Hospitalier d’Avignon, Avignon, France; 57 Groupe Hospitalier Diaconesses - Croix Saint Simon, Paris, France; 58 CHU Clermont-Ferrand - Hôpital Gabriel Montpied, Clermont Ferrand, France; 59 Hôpital d’Instruction Des Armées Percy, Clamart, France; 60 CHU Nancy - Hôpital Brabois, Vandoeuvre-Les-Nancy, France; 61 Centre Hospitalier de Vichy, Vichy, France; 62 Hopital Pierre Bérégovoy, Nevers, France; 63 Centre Hospitalier de Tarbes, Tarbes, France; 64 Hôpitaux Civils de Colmar – Hôpital Louis pasteur, Colmar, France; 65 CHU Charleroi - Hôpital Marie Curie, Brussels, Belgium; 66 Centre Hospitalier de Verdun Saint Mihiel, Saint Mihiel, France; 67 CH Eure-Seine - Hôpital d’Evreux-Vernon, Evreux, France; 68 Hôpital René Dubos, Pontoise, France; 69 APHP - Hôpital Lariboisière, Paris, France; 70 Centre Hospitalier de Saint-Brieuc, Saint-Brieuc, France; 71 Polyclinique Bordeaux Nord Aquitaine, Bordeaux, France; 72 HIA Sainte Anne, Toulon, France; 73 Grand Hôpital de L’Est Francilien, Meaux, France; 74 HIA Robert Picqué, Villenave d’Ornon, France; 75 Centre Hospitalier Fontainebleau, Fontainebleau, France; 76 Hôpital Universitaire de Genève, Geneva, Switzerland; 77 APHP - Hôpital Beaujon, Clichy, France; 78 Groupe Hospitalier Bretage Sud, Lorient, France; 79 Centre Hospitalier Intercommunal Toulon, La Seyne Sur Mer, France; 80 Centre Hospitalier de Dieppe, Dieppe, France; 81 CHU de Martinique, Fort-de-France, France; 82 Hôpital Fondation Adolphe de Rothchild, Paris, France; 83 APHP - Bichat Claude Bernard, Paris, France; 84 APHP - Hôpital Universitaire Paris Sud, Bicêtre, France; 85 APHP - Hôpital Européen Georges Pompidou, Paris, France; 86 APHP, GHU Henri Mondor, Créteil, France; 87 APHP - Hôpitaux Universitaires Henri Mondor, Créteil, France; 88 APHP - Hôpital Saint-Antoine, Paris, France; 89 APHP Hôpital Saint-Louis, Paris, France; 90 APHP – Hôpital Saint-Antoine, Paris, France; 91 APHP - Hôpital Raymond Pointcarré, Garches, France; 92 Centre Hospitalier Victor Dupouy, Argenteuil, France; 93 CHU Toulouse - Hôpital Rangueil, Toulouse, France; 94 Centre Hospitalier de Poissy, Poissy, France; 95 APHP - Hôpital Saint-Louis, Paris, France; 96 Clinique du MontLégia, CHC Groupe-Santé, Liège, Belgium; 97 CHU Saint-Denis, La Réunion, France; 98 Centre Hospitalier de Tourcoing, Tourcoing, France; 99 Centre Hospitalier Henri Mondor d’Aurillac, Aurillac, France; 100 Centre Hospitalier Saint Joseph Saint Luc, Lyon, France; 101 Centre Hospitalier de Polynésie Française, Polynésie, France; 102 Ramsay Générale de Santé, Hôpital Privé Jacques Cartier, Massy, France; 103 Centre Hospitalier Alpes Léman, Contamine Sur Arve, France; 104 Hospices Civils de Lyon - Hôpital de La Croix Rousse, Lyon, France; 105 Centre Cardiologique du Nord, Saint-Denis, France; 106 GHU - Hôpital Saint-Anne, Paris, France; 107 CHR Metz - Hôpital Mercy, Metz, France; 108 APHP - Hôpital Paul Brousse, Villejuif, France; 109 CHRU Nancy - Hôpital Central, Nancy, France; 110 Centre Hospitalier d’Ajaccio, Ajaccio, France; 111 Centre Hospitalier de Bourges, Bourges, France; 112 Centre Hospitalier de La Côte Basque, Bayonne, France; 113 CH Saint-Malo, Saint-Malo, France; 114 Centre Hospitalier de Mulhouse, Mulhouse, France; 115 Centre Hospitalier de Briançon, Briançon, France; 116 CHU Nice, Hôpital Pasteur 2, Nice, France; 117 Centre Hospitalier Des Pays de Morlaix, Morlaix, France; 118 Centre Hospitalier Valence, Valence, France; 119 Centre Hospitalier Niort, Niort, France; 120 Clinique du Val d’Or, Saint Cloud, France; 121 Centre Hospitalier de Béthune, Béthune, France; 122 Groupe Hospitalier Intercommunal de La Haute-Saône, Vesoul, France; 123 Clinique Saint-Martin, Caen, France; 124 Ramsay Générale de Santé, Clinique Convert, Bourg en Bresse, France; 125 Hôpital Victor Jousselin, Dreux, France; 126 Centre Hospitalier de Troye, Troye, France; 127 CHU de ROUEN-Hôpital Charles Nicolle, Rouen, France; 128 Centre Hospitalier Agen-Nérac, Agen, France; 129 APHP - Hôpital Louis Mourier, Colombes, France; 130 APHP – Hôpital Pitié-Salpêtrière, Paris, France; 131 Institut Mutualiste Montsouris, Paris, France; 132 CHU Besançon – Hôpital Jean Minjoz, Besançon, France; 133 APHP - Hôpital Universitaire Robert-Debré, Paris, France; 134 Médipôle Lyon-Villeurbanne, Vileurbanne, France; 135 APHP - Ambroise Paré, Boulogne-Billancourt, France; 136 CHU Amiens Picardie, Amiens, France; 137 Hôpital Nord-Ouest, Villefranche-Sur-Saône, France; 138 CH de Châlons en Champagne, Châlons en Champagne, France; 139 CHU Grenoble Alpes, Grenoble, France; 140 Hôpital Privé d’Antony, Antony, France; 141 Institut Arnault Tzanck, Saint Laurent du Var, France; 142 Centre Hospitalier d’ Angoulême, Angoulême, France; 143 Centre Hospitalier de Cahors, Cahors, France; 144 Centre Hospitalier de Carcassonne, Carcassonne, France; 145 CHU Nice – Hôpital L’Archet 2, Nice, France; 146 Hôpital Privé du Vert Galant, Tremblay-en-France, France; 147 Centre Hospitalier de Rambouillet, Rambouillet, France; 148 Hopitaux du Léman, Thonon Les Bains, France; 149 Centre Hospitalier Victor Jousselin, Dreux, France; 150 Hôpital Sainte Camille, Brie Sur Marne, France; 151 Hôpital d’instruction Des Armées Clermont-Tonnerre, Brest, France; 152 APHP - Hôpital Avicenne, Bobigny, France; 153 Groupement Hospitalier La Rochelle Ré Amis, La Rochelle, France; 154 Centre Hospitalier Intercommunal de Mont de Marsan Et du Pays Des Sources, Mont de Marsan, France; 155 Centre Hospitalier Départemental de Vendée, La-Roche-Sur-Yon, France; 156 Pôle Anesthésie-Réanimation, CHU Grenoble, La Tronche, France

**Keywords:** COVID-19, Outcome, Life-sustaining therapy, Ethical, Acute respiratory distress syndrome, Critical care

## Abstract

**Background:**

Limitations of life-sustaining therapies (LST) practices are frequent and vary among intensive care units (ICUs). However, scarce data were available during the COVID-19 pandemic when ICUs were under intense pressure. We aimed to investigate the prevalence, cumulative incidence, timing, modalities, and factors associated with LST decisions in critically ill COVID-19 patients.

**Methods:**

We did an ancillary analysis of the European multicentre COVID-ICU study, which collected data from 163 ICUs in France, Belgium and Switzerland. ICU load, a parameter reflecting stress on ICU capacities, was calculated at the patient level using daily ICU bed occupancy data from official country epidemiological reports. Mixed effects logistic regression was used to assess the association of variables with LST limitation decisions.

**Results:**

Among 4671 severe COVID-19 patients admitted from February 25 to May 4, 2020, the prevalence of in-ICU LST limitations was 14.5%, with a nearly six-fold variability between centres. Overall 28-day cumulative incidence of LST limitations was 12.4%, which occurred at a median of 8 days (3–21). Median ICU load at the patient level was 126%. Age, clinical frailty scale score, and respiratory severity were associated with LST limitations, while ICU load was not. In-ICU death occurred in 74% and 95% of patients, respectively, after LST withholding and withdrawal, while median survival time was 3 days (1–11) after LST limitations.

**Conclusions:**

In this study, LST limitations frequently preceded death, with a major impact on time of death. In contrast to ICU load, older age, frailty, and the severity of respiratory failure during the first 24 h were the main factors associated with decisions of LST limitations.

**Supplementary Information:**

The online version contains supplementary material available at 10.1186/s13054-023-04349-1.

## Background

In spring 2020, Europe experienced the first surge of the SARS-CoV-2 pandemic, leading to a large number of intensive care unit (ICU) admissions of severe COVID-19 patients requiring both prolonged mechanical ventilation and ICU stay, and placing an unprecedented strain on ICUs and healthcare systems. High mortality related to the most severe forms, including acute respiratory distress syndrome (ARDS), was initially reported as reaching 40% [1]. However, mortality rates decreased over time, with a regional variability observed between centres [2, 3]. Nationwide studies in the USA, the United Kingdom, France and Belgium investigated the reasons of the variability in mortality rates, with an emphasis on organizational aspects [2–7]. Importantly, findings suggested an increased COVID-19-related mortality when ICUs were overwhelmed. Facing an important surge of COVID-19 patients, facilities expanded their ICU capacities and, in some instances, scarcity of ventilators, ICU beds, staff resources, or drug use raised ethical dilemmas related to critical care resource allocation and triage [8, 9]. Ethical discussions are part of the daily process of care in the ICU where decisions to withdraw or withhold life-sustaining treatments (LST) are common. The recent Ethicus-2 study including 199 ICUs across four continents and 36 countries found an LST limitation cumulative incidence rate of 11.8% and an associated mortality of 71.9% and 88.5%, respectively, after withholding and withdrawing LST [10]. In addition, one half of deaths are preceded by LST decisions in the ICU [11, 12]. Age, comorbidities, and illness severity are considered important features when discussing LST limitations [13], but a substantial variability has been reported worldwide regarding the frequencies, modalities and timing of LST decisions, as well as between ICUs within countries [10, 14–16].

Before the COVID-19 pandemic, pressure on ICU capacity was suggested to influence both mortality [17, 18] and LST decisions [19]. However, few data have been reported to date on LST decisions in critically ill COVID-19 patients during the pandemic or when ICU capacities are facing an exceptional challenge. We sought to investigate the prevalence, cumulative incidence, timing and modalities of LST decisions in critically ill COVID-19 patients, as well as the individual and organizational factors associated with these decisions.

## Methods

### Study design and patients

We did an ancillary analysis of the COVID-ICU study, a multicentre, prospective, cohort study conducted in 164 ICUs across three European countries (France, Belgium, and Switzerland), which described outcomes and risk factors of 90-day mortality of critically ill COVID-19 patients [1]. The study was launched by the *Réseau Européen de recherche en Ventilation Artificielle* (REVA) and included all consecutive patients aged > 16 years admitted to participating ICUs with laboratory-confirmed SARS-CoV-2 infection between February 25 and May 4, 2020. The ethics committees of the French Intensive Care Society (CE-SRLF 20-23), Belgium (2020-294), and Switzerland (BASEC #2020-00704) approved the study according to regulations for each participating country. All patients or next of kin were informed that patient data would be anonymously included in the COVID-ICU database. Patients and relatives had the possibility to decline participation in the study. In that case, data were not collected. The study followed the STROBE statement for the reporting of observational studies [20].

All patients with laboratory-confirmed SARS-CoV-2 infection and available data regarding LST decisions and day-90 vital status were included in the study. Laboratory confirmation for SARS-Cov-2 was defined as a positive result of a real-time reverse transcriptase-polymerase chain reaction assay from either nasal or pharyngeal swabs, or lower respiratory tract samples.

### Data collection

Day 1 was defined as the first day when the patient was present in the ICU at 10 am. Each day, study investigators completed a standardized electronic case report form. Data collected included baseline demographic characteristics within the first 24 h after ICU admission (day 1), comorbidities, simplified acute physiology score (SAPS-II) [21], sequential organ failure assessment (SOFA) score [22], the clinical frailty scale score [23], date of first symptom/s, and ICU admission date. Local investigators documented the following information in a daily expanded dataset: presence of a respiratory support device (oxygen mask, high-flow nasal cannula, noninvasive or invasive mechanical ventilation); arterial blood gases; FiO_2_; PaO_2_/FiO_2_ ratio; use of neuromuscular blockers or corticosteroids (regardless of the indication and the dose); and standard laboratory parameters. Data were also collected on complications and organ dysfunction during the ICU stay, including acute kidney injury treated with renal replacement therapy, thromboembolic complications, ventilator-associated pneumonia and cardiac arrest, as well as detailed treatment limitation decisions.

If an LST limitation was decided upon during ICU stay, investigators were asked to record in detail the following items: cardiovascular support (vasopressors, do-not-resuscitate order); ventilatory support (invasive or non-invasive, intubation, tracheotomy, respiratory device settings, FiO_2_); renal replacement therapy; blood transfusion; enteral or parenteral nutrition; surgical emergency treatment; antibiotics; and intracranial pressure monitoring.

### Definitions

Geographical areas (hereafter referred to as “regions”) were set as the national administrative divisions, i.e., provinces for Belgium, departments for France, and cantons for Switzerland. To assess the strain on ICU capacities caused by the surge of COVID-19 patients, the ICU load was first computed at the regional level on a daily basis as:

$$\frac{{Number\;of\; ICU\; beds\; occupied\; by\; COVID{ - }19\; patients \;on\; a\; given\; day}}{Total\; number \;of\; baseline\; ICU\; beds\; before\; the\;pandemic }$$

This dynamic parameter was defined according to Bravata et al. [4] The ICU load at the patient level was finally defined as the mean ICU load in the region during the patient’s ICU stay. An ICU load of 100% reflected that all baseline ICU beds were occupied by COVID-19 patients, while an ICU load over 100% meant that the number of COVID-19 patients exceeded the baseline ICU hospitalization capacity. The number of baseline regional ICU beds before the pandemic and daily regional ICU bed occupancy during the first surge of the pandemic were based on data publicly available from official epidemiological reports on governmental websites including Public Health France and the French Ministry of Health, the Belgium Health Public Institute “Sciensano”, and the Swiss Federal Office of Public Health (see Additional file 1: Data Supplement, p 4). Treatment limitations were categorized as LST withholding or withdrawal, according to the decision recorded in the daily expanded dataset by local investigators (see Additional file 1: Data Supplement, p 5). A patient with a decision of LST withdrawal after an LST withholding decision was classified in the “LST withdrawal” group.

### Statistical analysis

Patients’ baseline characteristics, first 24-h in-ICU variables, treatments, organizational parameters, and ICU load at the patient level were described overall according to the following LST groups: (1) no LST; (2) LST withholding; and (3) LST withdrawal, whether or not preceded by an LST withholding decision. Continuous variables were described as medians (interquartile range [IQR]) and categorical variables as counts and percentages. Time to LST withholding and withdrawal decisions from ICU admission was estimated using a cumulative incidence function with ICU discharge and death during ICU stay as competing risks. Kaplan–Meier survival curves were plotted for the estimation of time to death from the first treatment limitation decision. In further analyses, the treatment limitation decision was dichotomized as LST withholding or withdrawal versus no limitation. Associations between variables and treatment limitation were estimated in a complete case analysis using a random intercept logistic regression model to account for the clustering of patients within centres. The following baseline variables obtained during the first 24 h in the ICU were included in the multivariable model and defined a priori (no statistical variable selection method was planned): age; gender; nursing home resident; clinical frailty scale score (non-frail [1–3], pre-frail [4], frail [≥ 5]); body mass index ≥ 30 kg/m^2^; diabetes; hypertension; chronic heart failure; ischemic cardiomyopathy; chronic respiratory disease; chronic kidney disease; immunodepression; past hematologic disease; time between first signs and ICU admission; ICU admission period; ICU load; SOFA cardiovascular component ≥ 3; SOFA renal component ≥ 3; and ARDS severity during the first 24 h in the ICU. A sensitivity analysis was performed in centres including ≥ 10 patients.

Heterogeneity in withholding/withdrawal decisions between centres was investigated using meta-analytical methods to combine proportions on a logit scale and evaluated using a likelihood ratio test. Variability between centres was assessed with the tau statistic (standard deviation of the random effect) [24]. This analysis was restricted to centres including 10 patients or more. Subgroup analyses were performed according to the number of patients included by centre (i.e., 10–29 patients, 30–49 patients, ≥ 50 patients) and in centres including at least 10 patients aged 75 years or over.

Analyses were performed on a complete case analysis with no missing data imputation. Statistical significance was set at the two-sided 0.05 value for all analyses. Analyses were computed with R software, version R-4.0.2 (R Foundation for Statistical Computing, Vienna, Austria, https://www.r-project.org).

### Role of the funding source

The funders of the study had no role in study design, data collection, data analysis, data interpretation, writing of the report, or the decision to submit for publication.

## Results

### Study population

**Fig. 1:**
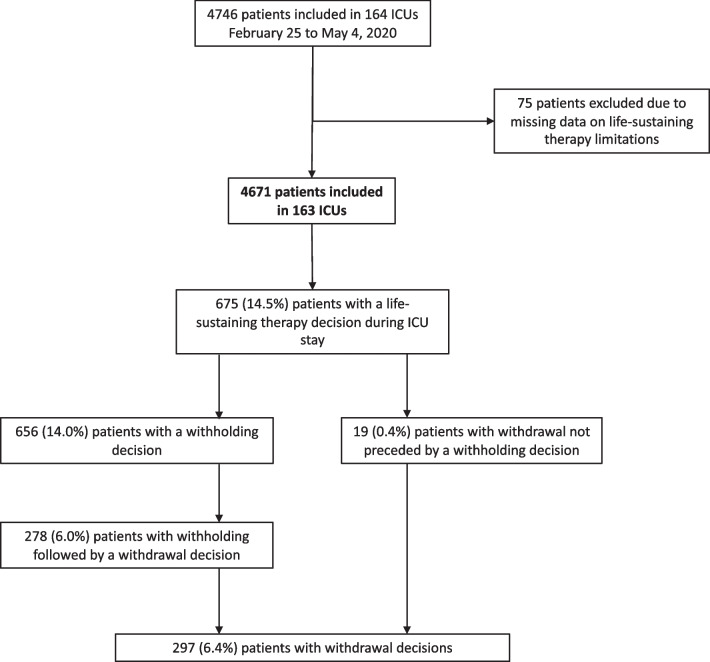
Flowchart of study population

Among the 4746 patients included in the study from February 25 to May 4, 2020, LST status recorded in the daily dataset report form was missing for 75 patients (Fig. 1). Hence, 4671 patients were included in the final analysis. Median age of patients was 63 (54–70) years, and 26% were women. Eighty-two percent had at least one comorbidity and the median clinical frailty scale score was 2 (2–3). ARDS severity was mild, moderate and severe in 24%, 49% and 28% of patients, respectively, and invasive mechanical ventilation was initiated for 2866 patients (61%) during the first 24 h. All baseline characteristics of the study population are shown in Table 1.

**Table 1:** Characteristics of the included study population according to LST withholding or withdrawing during ICU stay

Variable	Missing	All (*n* = 4671)	No limitation (*n* = 3996)	LST withholding (*n* = 378)	LST withdrawing (*n* = 297)
Age (years), median (IQR)	–	63 (54, 70)	61 (53, 69)	70 (63, 76)	71 (63, 78)
Female, *n* (%)	33	1191 (26)	1009 (25)	108 (29)	74 (25)
Healthcare worker, *n* (%)	83	160 (3)	149 (4)	7 (2)	4 (1)
Nursing home resident, *n* (%)	47	74 (2)	46 (1)	16 (4)	12 (4)
Obesity (BMI > 30 kg/m^2^), *n* (%)	336	1681 (39)	1457 (39)	134 (39)	90 (33)
SAPS II score, median (IQR)	403	37 (28, 50)	35 (27, 48)	44 (36, 56)	47 (35, 59)
SOFA score, median (IQR)	679	5 (3, 8)	4 (3, 8)	7 (4, 10)	7 (4, 10)
Hypertension, *n* (%)		2221 (48)	1831 (46)	224 (59)	166 (56)
Diabetes, *n* (%)		1271 (27)	1034 (26)	134 (35)	103 (35)
Coronary artery disease, *n* (%)		509 (11)	381 (10)	71 (19)	57 (19)
Chronic heart failure, *n* (%)		172 (4)	111 (3)	32 (8)	29 (10)
Chronic respiratory disease, *n* (%)	40	993 (21)	804 (20)	106 (28)	83 (28)
Immunodeficiency, *n* (%)	38	337 (7)	263 (7)	38 (10)	36 (12)
Chronic renal failure, *n* (%)	38				
Yes		324 (7)	247 (6)	45 (12)	32 (11)
Chronic dialysis		112 (2)	88 (2)	15 (4)	9 (3)
Hematological malignancy, *n* (%)		129 (3)	93 (2)	17 (4)	19 (6)
Clinical frailty scale score	465	2 (2–3)	2 (2–3)	3 (2–4)	3 (2–4)
Date of ICU admission, *n* (%)	167				
Before March 15, 2020		*n* = 295	225 (76)	39 (13)	31 (11)
From March 16 to 31, 2020		*n* = 2752	2343 (85)	227 (8)	182 (7)
From April 1 to 15, 2020		*n* = 1214	1072 (88)	75 (6)	67 (6)
After April 16, 2020		*n* = 243	208 (86)	22 (9)	13 (5)
Time between first symptoms and ICU admission	404				
< 4 days		*n* = 421	327 (78)	50 (12)	44 (10)
4–7 days		*n* = 1379	1138 (83)	134 (10)	107 (8)
≥ 8 days		*n* = 2467	2200 (89)	151 (6)	116 (5)
During the first 24 h, *n* (%)					
Standard oxygen therapy	94	1367 (29)	1202 (30)	103 (27)	62 (21)
Noninvasive ventilation	148	271 (6)	217 (5)	30 (8)	24 (8)
High-flow oxygen	162	873 (19)	767 (19)	67 (18)	39 (13)
Invasive mechanical ventilation	76	2866 (61)	2377 (60)	260 (69)	229 (77)
ARDS severity*, *n* (%)	412				
Mild		580 (24)	488 (24)	43 (19)	49 (24)
Moderate		1197 (49)	1000 (49)	109 (47)	88 (44)
Severe		677 (28)	534 (26)	80 (34)	63 (32)
Static compliance, mL/cmH_2_O	341	35 (29–43)	35 (29–43)	33.6 (28–42)	33.3 (28–42)

*LST* life-sustaining treatment, *IQR* interquartile range, *SAPS II* simplified acute physiology score II, *BMI* body mass index, *ICU* intensive care unit, *SOFA* sequential organ failure assessment, *ARDS* acute respiratory distress syndrome

*Data reported for patients under invasive mechanical ventilation

### Modalities of LST withholding and withdrawal

LST limitation decisions during ICU stay occurred for 675 (period prevalence = 14.5%) patients, including a withholding decision in 656 (period prevalence = 14.0%) and a withdrawal decision in 297 (period prevalence = 6.4%) patients. A withdrawal decision was mostly preceded by an LST withholding decision (278/297 [93.6%]) (Table 1). LST withholding frequently included several modalities, with 82% percent of patients presenting two or more modalities of withholding (see Additional file 1: Data Supplement, tables E1 and E2). A do-not-resuscitate order was the most frequent modality of LST withholding (86.6%), followed by limitations of renal replacement therapy initiation (62.8%) and an initiation or increase of vasopressors (60.2%). At the time of LST withholding decisions, patients who had a further decision of LST withdrawal included more treatment restrictions. LST withdrawal involved predominantly vasopressors (53.2%), renal replacement therapy (41.1%), and mechanical ventilation (31.6%). Extubation was decided in 24.9%. Of note, among all patients with a withholding decision (656), only 59 (9%) patients had solely a do-not-resuscitate order, and among these 59 patients, only 7 patients had finally a withdrawing.

**Table 2:** Patient characteristics associated with LST limitations

Factor	Crude odds ratio (95% confidence interval)	Adjusted odds ratio (95% confidence interval)	*P* value
Age (years)			< 0.001
[16–65)	–	–	
[65–75)	2.50 (1.93 to 3.23)	2.00 (1.51 to 2.65)	< 0.001
[75–91]	10.19 (7.64 to 13.59)	8.28 (5.94 to 11.54)	< 0.001
Female	1.07 (0.85 to 1.35)	0.91 (0.69 to 1.20)	0.507
Nursing home resident	3.86 (2.11 to 7.06)	1.41 (0.65 to 3.07)]	0.381
Clinical frailty scale score			< 0.001
[1–3]	–	–	
(3–4]	3.28 (2.42 to 4.45)	1.61 (1.11 to 2.32)	0.012
(4–10]	6.21 (4.27 to 9.03)	3.03 (1.89 to 4.83)	< 0.001
Body mass index ≥ 30 kg/m^−2^	0.88 (0.71 to 1.09)	0.92 (0.71 to 1.19)	0.529
Hypertension	1.81 (1.47 to 2.23)	1.06 (0.82 to 1.37)	0.647
Diabetes	1.64 (1.32 to 2.04)	1.39 (1.06 to 1.81)	0.016
Coronary artery disease	2.59 (1.96 to 3.41)	1.31 (0.93 to 1.85)	0.119
Chronic heart failure	4.25 (2.85 to 6.33)	1.80 (1.11 to 2.91)	0.016
Chronic respiratory disease	1.65 (1.31 to 2.09)	1.26 (0.95 to 1.65)	0.105
Immunodeficiency	1.61 (1.13 to 2.30)	1.44 (0.95 to 2.19)	0.086
Chronic renal failure			0.573
None	–	–	
Yes w/o dialysis	2.32 (1.65 to 3.24)	1.13 (0.74 to 1.73)	0.578
Chronic dialysis	2.21 (1.27 to 3.86)	1.43 (0.72 to 2.86)	0.311
Hematological malignancy	1.99 (1.32 to 3.01)	1.77 (1.09 to 2.88)	0.020
Period of admission			0.106
Before March 15, 2020	–	–	
March 16 to 31, 2020	0.51 (0.35 to 0.73)	0.73 (0.47 to 1.14)	0.164
April 1 to 15, 2020	0.36 (0.24 to 0.55)	0.55 (0.33 to 0.92)	0.021
After April 16, 2020	0.47 (0.26 to 0.83)	0.58 (0.29 to 1.13)	0.110
Time since 1st symptom			0.003
< 4 days	–	–	
4–7 days	0.78 (0.56 to 1.08)	1.02 (0.70 to 1.50)	0.902
≥ 8 days	0.42 (0.30 to 0.58)	0.67 (0.46 to 0.98)	0.041
ICU load (%)			0.010
≤ 100	–	–	
(100–150]	0.58 (0.43 to 0.78)	0.70 (0.50 to 0.99)	0.042
(150–200]	0.47 (0.34 to 0.63)	0.63 (0.44 to 0.91)	0.014
> 200	0.57 (0.41 to 0.80)	1.14 (0.75 to 1.73)	0.533
First 24-h respiratory failure severity			< 0.001
Not intubated	–	–	
Mild ARDS PF (200–600]	1.64 (1.21 to 2.23)	1.89 (1.27 to 2.81)	0.002
Moderate ARDS PF (100–200]	1.80 (1.37 to 2.37)	2.03 (1.40 to 2.94)	< 0.001
Severe ARDS PF (0–100]	3.23 (2.37 to 4.41)	3.61 (2.42 to 5.37)	< 0.001
SOFA cardiovascular ≥ 3	1.84 (1.49 to 2.27)	1.11 (0.83 to 1.48)	0.499
SOFA renal ≥ 3	1.97 (1.39 to 2.80)	1.39 (0.90 to 2.17)	0.141

*LST* life-sustaining treatment, *ICU* intensive care unit, *SOFA* sequential organ failure assessment, *ARDS* acute respiratory distress syndrome

**Fig. 2:**
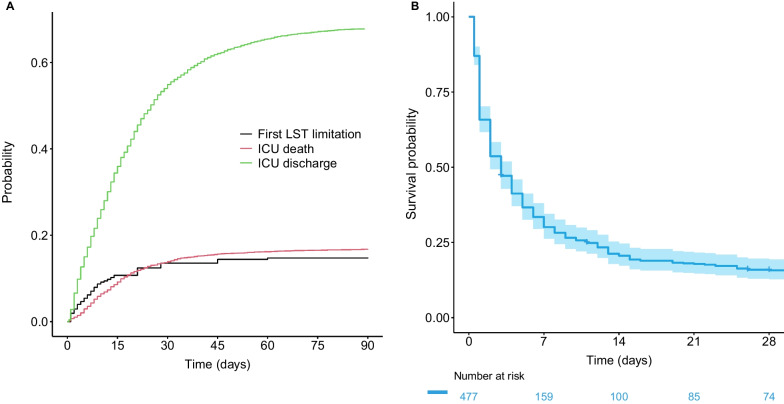
**A** Cumulative incidence plot of time from ICU admission to first LST limitation decision, and **B** survival probability after LST withholding or withdrawing decisions within 14 days after ICU admission

At 28 and 90 days, the cumulative incidence of LST limitation in patients with complete date data (4549/4671) was 12.4% and 14.8%, respectively (Fig. 2A). Decisions on LST limitations were taken at a median of 8 days (range 3–21) after ICU admission. The cumulative incidence of LST limitation in patients with complete data for all variables (3051/4671) is shown in Figure E1 (see Additional file 1: Data Supplement). The results were similar between these two cohorts.

### Factors associated with LST limitations

Median ICU load was 126% (range 71–187). ICU load distribution at the patient level and according to LST categories are presented in Figures E2 and E3 (see Additional file 1: Online Data Supplement). The multivariable model included 3051 patients (Table 2). Age, clinical frailty scale score, and first 24-h respiratory failure severity were independently associated with a decision of LST limitation. Importantly, the odds ratios of the age categories ≥ 65 and ≤ 75 years and ≥ 75 years were 2.00 (1.51–2.64) and 8.30 (5.95–11.6), respectively, compared to patients aged < 65 years (*p* < 0.001). The decision of LST limitation was significantly associated with pre-frail and frail status compared to non-frail patients, with odds ratios of 1.61 (1.11–2.32) and 3.02 (1.89–4.82), respectively (*p* < 0.001). By contrast, an ICU load over 100% was associated with a decreased probability of LST limitation, but time to LST decision did not differ according to ICU load category. A sensitivity analysis yielded similar results when omitting centres including < 10 patients (data not shown).

Centre characteristics, adjunct measures during ICU stay according to LST limitation status, and time to LST decision according to ICU load category are presented in Tables E3, E4 and E5, respectively (see Additional file 1: Online Data Supplement).

### Variability of LST limitations between centres

**Fig. 3:**
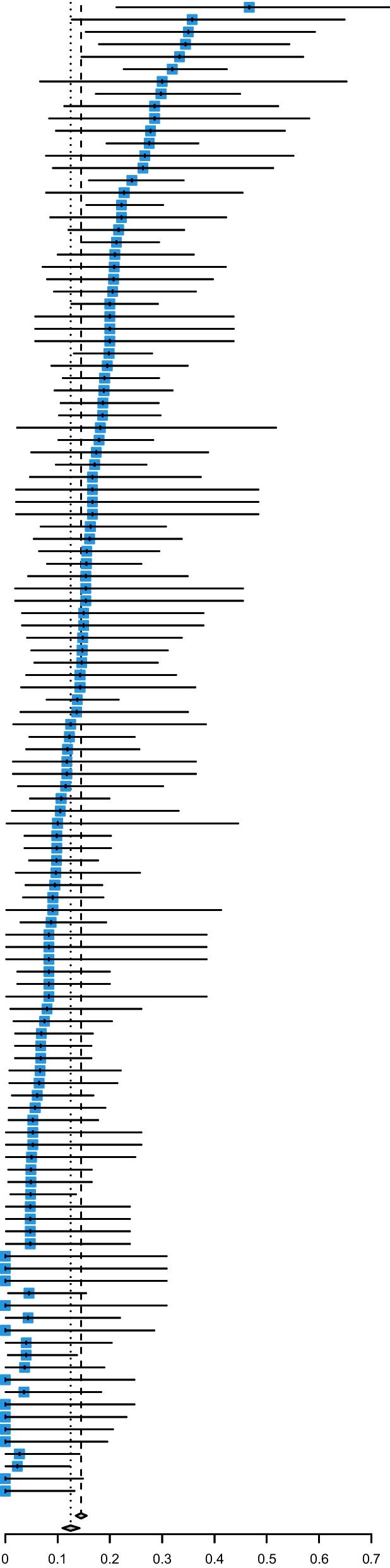
Distribution of estimated prevalence of life-sustaining treatment limitations according to centres

Of the 163 participating centres, 121 included 10 patients or more, representing a total of 4492 patients. The estimated overall proportion of patients with an LST limitation was 12.5% (95% CI 11.0–14.2; Fig. 3). There was a significant heterogeneity between centres, with a tau of 0.539 (likelihood-ratio test *p* value < 0.001). Hence, it was expected that the prevalence of LST limitations in 95% of centres would lie within 4.7% and 29.1%. Similar results were observed in the subpopulation of patients aged ≥ 75 years and regardless to the number of patients included per centre (see Additional file 1: Tables E6, E7, Figure E4).

### Outcomes

Overall, 1347 patients (29%) died within 90 days of follow-up after ICU admission. An LST withholding or withdrawal decision during ICU stay preceded death in 561 patients (42%). Of the 675 patients who experienced treatment limitation during their ICU stay, 561 (83%) died within 90 days of follow-up. In-ICU death occurred for 279 (74%) and 282 (95%) patients, respectively, after LST withholding and withdrawal. Survival time after the first treatment limitation decision was evaluated in patients with an LST decision within 14 days after ICU admission. Median survival time was 3 days (range 1–11), with a 28- and 60-day survival after a first limitation of 15.7% (95% CI 12.8–19.4) and 14.2% (95% CI 11.4–17.7), respectively (Fig. 2B).

## Discussion

This European multicentre study of 4671 patients provides the most exhaustive descriptive analysis to date on LST limitations in patients admitted to the ICU during the first surge of the COVID-19 pandemic. The global period prevalence of in-ICU LST limitation decisions was 14.5%, with an important variability between centres. However, this variability was not related to the patient and/or centre characteristics analyzed in the study. Age, clinical frailty scale score, and respiratory severity were the patient characteristics most associated with decisions of LST limitations. Interestingly, while ICU load reflected an overwhelming surge of COVID-19 patients admitted to the ICU, the strain on ICU capacities was associated with a decreased probability of LST limitation. Not unexpectedly, decisions of LST withholding and withdrawal were followed by high short-term mortality and frequently preceded in-ICU death.

With regards to previously published data on LST limitations in the ICU [10, 14, 15], our findings reflected a similar incidence rate and patient characteristics taken into account for ethical decision-making. A 14.5% prevalence of LST limitations is consistent with the recently reported incidence of 11.8% of all-cause ICU admissions reported in the worldwide Ethicus-2 study [10]. Older age and illness severity at ICU admission was associated with limitations of LST as already demonstrated in a general population of critically ill patients [14]. However, the frailty score was only found to be associated with decisions of LST limitations in the ICU in the very elderly (≥ 80 years) [13]. To the best of our knowledge, this is the first time that factors associated with LST limitations in a setting of an overwhelming surge of critically ill patients or in a subpopulation of acute hypoxic respiratory failure patients have been identified.

The important (up to six-fold) variability of the prevalence of LST limitations observed between centres in a homogeneous population of severe COVID-19 patients represents a significant and original result of our study. Interestingly, this variability was not explained by patient or organizational characteristics of the centres. Indeed, between-ICU variability in ethical decision-making in the same range has already been reported in nationwide and international studies [10, 16, 25]. Some factors unrelated to individual characteristics have been identified as associated with this variability. For example, the frequency of LST limitations is higher in countries with a high gross domestic product and lower in countries where religion is important [13]. In addition, there is currently no uniform approach to the LST limitation decision-making process that could take into account all individual, relatives and social determinants, particularly in a new disease. Therefore, variability between ICUs possibly reflects differences in institutional policies [25].

A significant variability in mortality rates was reported in several nationwide studies during the COVID-19 pandemic, with some highlighting an increased mortality rate when ICU resources were strained, but without any clear explanation of the mechanism involved [2–7]. A retrospective study of 9891 patients who died in the ICU suggested the positive association of strain on ICU capacities and a shorter time to end-of-life decision-making [19]. In the context of important ethical discussions regarding the allocation of critical care resources during the first surge of the pandemic, it appeared important to investigate the effect of ICU load on LST limitation decisions. Using the parameter proposed by Bravata et al.,[4] we were able to calculate this marker reflecting strain on regional ICU resources from pre-pandemic baseline capacities and daily ICU bed occupancy data at the patient level. Despite an increased median of 126%, ICU load was associated with a lower treatment limitation probability. However, we were unable to investigate if this result was due to triage before ICU admission. Another unexplored hypothesis to explain the increased mortality in regions of high ICU strain could be related to understaffing leading to suboptimal practices, with an impact on adverse events [18].

Of note, there are few data on LST limitations in the literature reporting an epidemiological analysis of severe COVID-19 patients during the pandemic [26], apart from the COVIP European study, which described the characteristics of elderly patients admitted to ICUs [27]. The latter study reported a higher incidence rate of LST limitation at day 30 in COVID-19 patients compared to non-COVID-19 patients. LST limitation was associated with the frailty scale score[28] and the COVID-19 incidence rate [29], thus suggesting that decisions of LST limitations could potentially be influenced by pressure on the healthcare system. Unfortunately, the authors did not explicitly investigate the relationship of LST limitations with strain on ICU resources.

The high mortality following decisions of LST withholding and withdrawal in our cohort of severe COVID-19 patients is similar to rates reported in the pre-pandemic literature [10]. However, when added to the prevalence of LST limitations observed and the unexplained variability across centres, these results emphasize the need to improve the reporting of LST limitations in randomized, controlled trials of COVID-19 patients managed in the ICU. Indeed, knowledge of LST decisions is of importance when assessing mortality or short-term endpoints, such as organ support-free days or duration, as both the modality and timing of LST limitations undoubtedly impact on mortality. Messika et al. demonstrated that LST limitations were rarely reported in randomized, controlled trials in critical care and that an imbalance between two groups concerning the proportion of LST decisions may affect results, particularly in open design trials [30]. To date, no randomized, controlled trial including COVID-19 patients in the ICU setting has reported rates and timing of LST limitations or proposed the standardization of treatment limitation decisions.

The strength of our study lies in the detailed description of decisions of LST limitations in the ICU during the first surge of the COVID-19 pandemic. However, the study has several limitations. First, we focused on decisions of LST limitation during ICU stay, but we are not able to provide data on triage before ICU admission. Second, our analysis was restricted to patients admitted to the ICU during the first surge. Considering likely changes of ICU practices after the first wave and a steep learning curve in the context of this new disease, we cannot exclude a subsequent different prevalence of LST limitations. However, some of our findings in the particular context of COVID-19 confirmed previous reports in a general ICU patient population. Third, as reported in the tables, some variables have missing data due to an important workload for intensivists during the first surge of the pandemic, which prevented the completion of research case report forms. Fourth, we did not investigate the variability of the timing of LST limitations between centres. Finally, we recognize that the method used to calculate the ICU load parameter over the entire patient ICU stay may have underestimated the exact load of care on a given day. Considering that all LST limitation decisions have been made during the ICU stay, we believe that this approach has limited the temporal delay between ICU load and LST limitation decision. In addition, we made the assumption that ICU load was the best parameter to assess ICU strain, with the hypothesis that the COVID-19-related ICU load was inferior to 100% of baseline ICU bed occupancy in France and Belgium before March 19, 2020, which is the date from when daily ICU occupancy data were communicated.

## Conclusions

In this multicentre observational study, older age, frailty, and the severity of respiratory failure during the first 24 h were the main factors associated with decisions of LST limitations. Our results did not support the association between ICU load and higher mortality. Importantly, our results showed very significant differences in LST limitation rates between centres. LST limitations frequently preceded death, with a major impact on time of death, and this should be reported in future studies evaluating ICU mortality with severe COVID-19 or critically ill patients.

## Supplementary Information

**Additional file 1**. Data collection. Epidemiological data used for ICU load calculation. Definitions of withholding and withdrawal of life-sustaining therapies (LST). Statistical analysis: multivariable model and list of the variables included in the model.** Table E1**. Modalities of LST withholding and withdrawal in the study population.** Table E2**. Modalities of LST withholding according to a further (or not) LST withdrawal decision.** Table E3**. Centre characteristics at the patient level.** Table E4**. Adjunct measures during ICU stay according to LST limitation status.** Table E5**. Time to LST decision from ICU admission according to ICU load category.** Figure E1**. (A) Cumulative incidence plot of time from ICU admission to first LST limitation decision, and (B) Survival probability after LST withholding or withdrawing decisions within 14 days after ICU admission involving only patients with complete data (3051 patients).** Figure E2**. Distribution of ICU load at the patient level.** Figure E3**. ICU load (%) according to LST categories. Subgroup analysis by centre size, and in patients aged ≥75 years.** Table E6**. Expected prevalences estimated by the multivariate model according to the number of patients in the centre.** Table E7**. Prevalence of decisions of LST limitations in patients aged ≥ 75 years.** Figure E4**. Forest plot of prevalences of decisions of LST limitations in the 15 centres with ≥ 10 patients aged ≥75 years.

**Additional file 2**. Participating Sites and COVID–ICU Investigators.

## Data Availability

The datasets used and/or analysed during the current study are available from the corresponding author on reasonable request.

## Abbreviations

ARDS: Acute respiratory distress syndrome
ICU: Intensive care unit
IQR: Interquartile range
LST: Life-sustaining therapies
REVA: Réseau Européen de recherche en Ventilation Artificielle
SAPS-II: Simplified acute physiology score II
SOFA: Sequential organ failure assessment
