# Supplementary material for: Limitation of life-sustaining therapies in critically ill patients with COVID-19: a descriptive epidemiological investigation from the COVID-ICU study
Source: Crit Care. 2023 Mar 11;27:103. doi: 10.1186/s13054-023-04349-1 (PMC10006561; doi:10.1186/s13054-023-04349-1)
Supplement: Supplementary file 1 — Additional file 1. Data collection. Epidemiological data used for ICU load calculation. Definitions of withholding and withdrawal of life-sustaining therapies (LST). Statistical analysis: multivariable model and list of the variables included in the model. Table E1. Modalities of LST withholding and withdrawal in the study population. Table E2. Modalities of LST withholding according to a further (or not) LST withdrawal decision. Table E3. Centre characteristics at the patient level. Table E4. Adjunct measures during ICU stay according to LST limitation status. Table E5. Time to LST decision from ICU admission according to ICU load category. Figure E1. (A) Cumulative incidence plot of time from ICU admission to first LST limitation decision, and (B) Survival probability after LST withholding or withdrawing decisions within 14 days after ICU admission involving only patients with complete data (3051 patients). Figure E2. Distribution of ICU load at the patient level. Figure E3. ICU load (%) according to LST categories. Subgroup analysis by centre size, and in patients aged ≥75 years. Table E6. Expected prevalences estimated by the multivariate model according to the number of patients in the centre. Table E7. Prevalence of decisions of LST limitations in patients aged ≥ 75 years. Figure E4. Forest plot of prevalences of decisions of LST limitations in the 15 centres with ≥ 10 patients aged ≥75 years. [file 13054_2023_4349_MOESM1_ESM.docx]

**Online Data Supplement**

**Limitation of life-sustaining therapies in critically-ill patients with COVID-19: a descriptive epidemiological investigation from the COVID-ICU study**

Mikhael Giabicani, MD^1,2*^, Christophe Le Terrier, MD^3*^, Antoine Poncet, MSc^4,5^, Bertrand Guidet, MD, PhD^6^, Jean-Philippe Rigaud, MD^7^, Jean-Pierre Quenot, MD, PhD^8^, Marie-France Mamzer, MD, PhD^2,9^, Jérôme Pugin, MD^3^, Emmanuel Weiss, MD, PhD^1^, Simon Bourcier, MD, PhD^3^on behalf of the COVID-ICU study investigators

***** Contributed equally

^1^ Department of Anaesthesiology and Critical Care, Beaujon Hospital, DMU Parabol, AP-HP Nord, Paris, France

^2^ Centre de Recherche des Cordeliers, Sorbonne Université, Université Paris Cité, Inserm, Laboratoire ETREs, Paris, France.

^3^ Division of Intensive Care, Geneva University Hospitals and the University of Geneva Faculty of Medicine, Geneva, Switzerland

^4^ Clinical Research Centre, Faculty of Medicine, University of Geneva, Geneva, Switzerland

^5^ Division of Clinical Epidemiology, Department of Health and Community Medicine, University Hospitals of Geneva, Geneva, Switzerland

^6^ Service de Réanimation Médicale, Assistance Publique-Hôpitaux de Paris, Hôpital Saint-Antoine, Paris, France.

^7^ Réanimation polyvalente, Centre Hospitalier de Dieppe, Dieppe, France

^8^ Department of Intensive Care, François Mitterrand University Hospital, Dijon, France

^9^ Unité Fonctionnelle d’Ethique Médicale, Hôpital Necker-Enfants malades, APHP, Paris, France

**List of additional files**

**Data collection**

**Epidemiological data used for ICU load calculation**

**Definitions of withholding and withdrawal of life-sustaining therapies (LST)**

**Statistical analysis: multivariable model and list of the variables included in the model**

**Table E1**. Modalities of LST withholding and withdrawal in the study population

**Table E2**. Modalities of LST withholding according to a further (or not) LST withdrawal decision

**Table E3**. Centre characteristics at the patient level

**Table E4.** Adjunct measures during ICU stay according to LST limitation status

**Table E5.** Time to LST decision from ICU admission according to ICU load category

**Figure E1.** (A) Cumulative incidence plot of time from ICU admission to first LST limitation decision, and (B) Survival probability after LST withholding or withdrawing decisions within 14 days after ICU admission involving only patients with complete data (3051 patients)

**Figure E2**. Distribution of ICU load at the patient level

**Figure E3**. ICU load (%) according to LST categories

**Subgroup analysis by centre size, and in patients aged ≥75 years**

**Table E6.** Expected prevalences estimated by the multivariate model according to the number of patients in the centre

**Table E7.** Prevalence of decisions of LST limitations in patients aged ≥ 75 years

**Figure E4.** Forest plot of prevalences of decisions of LST limitations in the 15 centres with ≥ 10 patients aged ≥75 years

**Data collection**

Day1 was defined as the first day when the patient was in the intensive care unit (ICU) at 10 am. Each day, the study investigators completed a standardized electronic case report form. Baseline information collected at ICU admission were: age; sex; body mass index; active smoker; simplified acute physiology score (SAPS II)^1^; sequential organ failure assessment (SOFA) score ^2^; comorbidities; immunodeficiency (if present); clinical frailty scale score^3^; date of first symptom; and dates of hospital and ICU admissions.

The clinical frailty scale is an ordinal hierarchical scale with a 1 to 9 ranking: 1, very fit; 2, well; 3, managing well; 4, vulnerable; 5, mildly frail; 6, moderately frail; 7, severely frail; 8, very severely frail; and 9 terminally ill. Eventually, patients were categorised into three clinical frailty score groups: 1 to 3 (fit); 4 (vulnerable, but not frail); and 5 to 9 (frail) for our analyses. Immunodeficiency was defined as hematological malignancies, an active solid tumor or having received specific anti-tumor treatment within a year, solid organ transplant; human immunodeficiency virus or immunosuppressants; chronic respiratory disease included asthma, chronic obstructive pulmonary disease, restrictive pulmonary disease, or apnea. The mode of respiratory support was also collected: invasive/non-invasive mechanical ventilation; oxygen mask/high flow nasal oxygen; FiO_2_, PaO_2_/FiO_2_ ratio; and use of neuromuscular blockades and corticosteroids (regardless of the indication and the dose). For patients who received standard oxygen therapy, day 1 PaO_2_/FiO_2_ was calculated by converting O_2_ flow to the estimated FiO_2_.^4^ We also collected data on complications and organ dysfunction during ICU stay, including acute renal failure requiring renal replacement therapy, thromboembolic complications (distal venous thrombosis or proven pulmonary embolism by either pulmonary CT angiography or cardiac echography), ventilator-associated pneumonia, and cardiac arrest. Ventilator-associated pneumonia was defined by either bronchoalveolar lavage cultures growing ≥10^4^cfu/mL, blind-protected, specimen brush distal sampling growing ≥10^3^cfu/mL, or endotracheal aspirates growing ≥10^6^cfu/mL.

If an LST limitation was decided upon during ICU stay, investigators were asked to record in detail the following items: cardiovascular support (vasopressors, do-not-resuscitate order); ventilatory support (invasive or non-invasive, intubation, tracheotomy, respiratory device settings, FiO_2_); renal replacement therapy; blood transfusion; enteral or parenteral nutrition; surgical emergency treatment; antibiotics; and intracranial pressure monitoring.

**Epidemiological data used for ICU load calculation**

Daily regional ICU bed occupancy data was based on data publicly available from official epidemiological reports on governmental websites: France: Public Health France agency <https://www.data.gouv.fr/fr/datasets/donnees-hospitalieres-relatives-a-lepidemie-de-covid-19/>; Belgium: Health Public Institute Sciensano and Epistat platform (<https://epistat.sciensano.be/Data/COVID19BE_HOSP.csv>); Swiss Confederation: Covid-19 portal (<https://www.covid19.admin.ch/fr/overview>).

ICU bed occupancy was not available before March 19, 2020, for France and Belgium due to the delay in setting up the monitoring systems and to the absence of a COVID-19 related strain on ICU capacities at the early phase of the first wave. ICU load before this date was consequently considered as ≤100%.

Numbers of baseline regional ICU beds before the pandemic were obtained in France from the Ministry of Health website (<https://drees.solidarites-sante.gouv.fr/article/nombre-de-lits-de-reanimation-de-soins-intensifs-et-de-soins-continus-en-france-fin-2013-et>), and in Belgium from the Health Public Institute Sciensano (<https://covid-19.sciensano.be/fr/covid-19-situation-epidemiologique>). In Belgium, 60% of the total number of recognized ICU beds were reserved for COVID-19 patients as reported by Sciensano and Taccone et al^5^ and was therefore the baseline number of beds taken into account for ICU load calculation. In the canton of Geneva of the Swiss Confederation, data were provided by the authors of this article affiliated to the Division of Intensive Care of Geneva University Hospitals (CLT, JP, SB).

**Definitions of withholding and withdrawal of life-sustaining therapies (LST)**

**LST withholding modalities** included: a do-not-resuscitate-order; renal replacement therapy; vasopressors; no emergency surgical treatment; blood transfusion; FiO_2_; intubation; antibiotherapy; intracranial pressure monitoring; tracheotomy; limitation of ventilator settings (mode); feeding (enteral or parenteral); and non-invasive mechanical ventilation.

**LST withdrawal modalities** included: vasopressors; renal replacement therapy; invasive mechanical ventilation; extubation; and non-invasive mechanical ventilation.

**Statistical analysis: multivariable model and list of the variables included in the model**

The treatment limitation decision was dichotomized as LST withholding or withdrawal versus no limitation. Associations between variables and treatment limitation were estimated in a complete case analysis using a random intercept logistic regression model to account for the clustering of patients within centres.

The following baseline variables obtained during the first 24 h in the ICU were included in the multivariable model and defined *a priori* (no statistical variable selection method was planned): age; gender; nursing home resident; clinical frailty scale (non-frail [1–3], pre-frail [4], frail [≥5]); body mass index ≥30 kg/m^2^; diabetes; hypertension; chronic heart failure; ischemic cardiomyopathy; chronic respiratory disease; chronic kidney disease; immunodepression; past hematologic disease; time between first signs and ICU admission; ICU admission period; ICU load; SOFA cardiovascular component ≥3; SOFA renal component ≥3; and ARDS severity during the first 24 h in the ICU.

**Table E1.** Modalities of withholding and withdrawal of LST in the study population

| **LST withholding (N=656)** | **N** |
| --- | --- |
| Do-not-resuscitate-order | 568 |
| Renal replacement therapy | 412 |
| Vasopressors | 395 |
| No emergency surgical treatment | 299 |
| Blood transfusion | 240 |
| FiO_2_ | 209 |
| Intubation | 205 |
| Antibiotherapy | 178 |
| Intracranial pressure monitoring | 165 |
| Tracheotomy | 140 |
| Limitation of ventilator settings (mode) | 136 |
| Feeding (enteral or parenteral) | 136 |
| Non-invasive mechanical ventilation | 102 |
| Missing | 24 |
| **LST withdrawal (N=297)** | **N** |
| Vasopressors | 158 |
| Renal replacement therapy | 122 |
| Invasive mechanical ventilation | 94 |
| Extubation | 74 |
| Non-invasive mechanical ventilation | 44 |
| Missing | 41 |

**Table E2.** Modalities of LST withholding according to a further (or not) LST withdrawal decision

|  | **Withholding followed by withdrawal** | |
| --- | --- | --- |
| **Modality of LST withholding** | **No (N=378)** | **Yes (N=278)** |
| Do-not-resuscitate-order | 316 (88) | 252 (93) |
| Renal replacement therapy | 207 (57) | 205 (76) |
| Vasopressors | 194 (54) | 201 (74) |
| Intubation | 104 (29) | 101 (37) |
| No emergency surgical treatment | 102 (28) | 197 (73) |
| Blood transfusion | 63 (17) | 177 (65) |
| Fraction of inspired oxygen | 57 (16) | 152 (56) |
| Tracheotomy | 55 (15) | 85 (31) |
| Intracranial pressure monitoring | 52 (14) | 113 (42) |
| Limitation of ventilator settings (mode) | 49 (14) | 87 (32) |
| Antibiotherapy | 30 (8) | 148 (55) |
| Non-invasive mechanical ventilation | 28 (8) | 74 (27) |
| Feeding (enteral or parenteral) | 13 (4) | 123 (45) |
| Missing | 17 | 7 |

**Table E3.** Centre characteristics at the patient level

| **Variable** | **Missing** | **All (n=4671)** | **No limitation (n=3996)** | **Withholding (n=378)** | **Withdrawal (n=297)** |
| --- | --- | --- | --- | --- | --- |
| **Hospital type**, n (%) | 57 |  |  |  |  |
| University |  | 2704 (59) | 2303 (58) | 222 (59) | 179 (61) |
| Public regional |  | 1446 (31) | 1229 (31) | 123 (33) | 94 (32) |
| Semi-private |  | 175 (4) | 154 (4) | 10 (3) | 11 (4) |
| Private |  | 289 (6) | 259 (7) | 20 (5) | 10 (3) |
| Number of beds in the hospital,  median (IQR) | 888 | 730 (500, 1200) | 717 (500, 1200) | 740 (550, 1038) | 734 (558, 1295) |
| Number of ICU beds in the centre | 634 | 24 (16, 32) | 23 (16, 32] | 26 (19, 34] | 24 (17, 32] |
| **In ICU: number of**, median (IQR) |  |  |  |  |  |
| Attending physicians | 734 | 8 (6, 12) | 8 (6, 12) | 8 (6, 12) | 8 (7, 14) |
| Residents | 734 | 8 (5, 10) | 8 (5, 10) | 8 (6, 10) | 8 (7, 10) |
| Nurses | 854 | 44 (33, 63) | 42 (33, 63) | 50 (34, 70) | 49 (34, 74) |
| Nursing auxiliaries | 938 | 28 (20, 36) | 28 (19, 36) | 30 (20, 38) | 28 (20, 36) |

**Table E4.** Adjunct measures during ICU stay according to LST limitation status

| **Variable** | **Missing*** | **All (n= 3686)** | **No limitation (n=3094)** | **Withholding (n=319)** | **Withdrawal (n=273)** |
| --- | --- | --- | --- | --- | --- |
| Tracheotomy | 1 | 568 (28) | 496 (26) | 53 (56) | 19 (61) |
| Prone position | 77 | 2431 (67) | 1986 (66) | 244 (77) | 201 (74) |
| Continuous neuromuscular blockers | 90 | 3135 (87) | 2609 (87) | 279 (88) | 247 (91) |
| Inhaled nitric oxide | 94 | 643 (18) | 494 (16) | 80 (25) | 69 (26) |
| Corticosteroids | 23 | 1506 (41) | 1205 (39) | 159 (50) | 142 (52) |
| Extracorporeal membrane  oxygenation | 30 | 342 (9) | 294 (10) | 22 (7) | 26 (10) |

*Missing data among patients under invasive mechanical ventilation.

**Table E5.** Time to LST decision from ICU admission according to ICU load category

| **ICU load (%)** | **n=951** | **Time to LST decision (days) median (IQR)** |
| --- | --- | --- |
| ≤ 100 | 308 | 8 (3 – 21) |
| (100-150] | 122 | 7 (3 – 13) |
| (150-200] | 115 | 9 (4.5 – 21) |
| > 200 | 98 | 8 (4 – 19) |


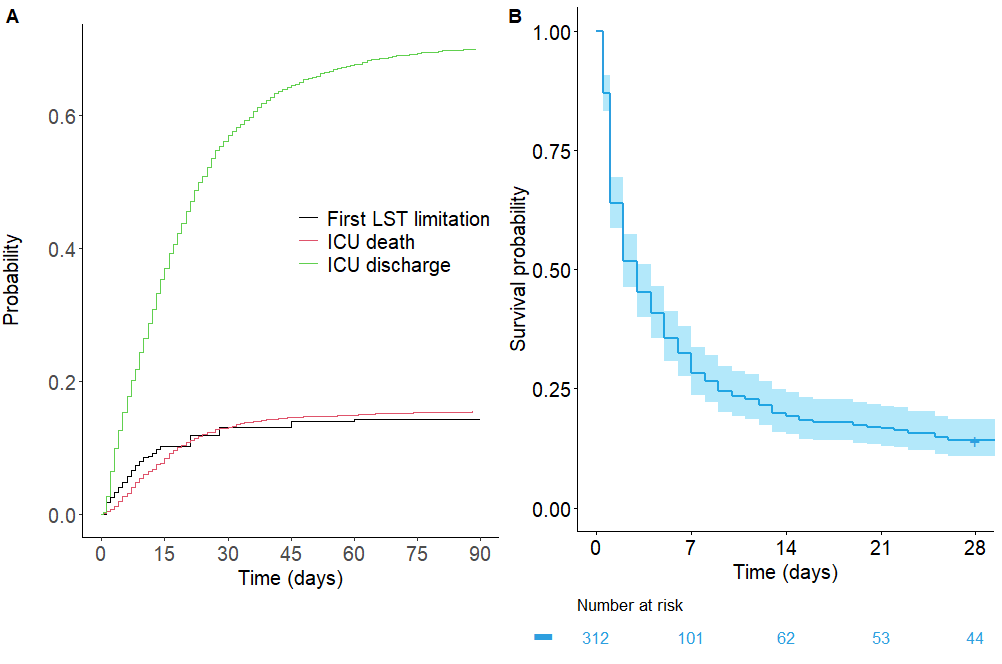
**Figure E1.** (A) Cumulative incidence plot of time from ICU admission to first LST limitation decision, and (B) survival probability after LST withholding or withdrawing decisions within 14 days after ICU admission involving only patients with complete data (3051 patients)

**Figure E2.** Distribution of ICU load at the patient level

Average % of occupied beds in the regions during ICU stay

**Figure E3.** ICU load (%) according to LST categories

None Withholding Withdrawal

LST

**Subgroup analysis by centre size, and in patients aged ≥75 years**

**Centre size**

Similar results were observed in a subgroup analysis based on the number of patients per centre (10 to 29 patients included = “small” participating centre; 30 to 49 = “medium”; 50 or more = “large”).

**Table E6.** Expected prevalences estimated by the multivariate model according to the number of patients in the centre

| **Centre size (number of patients)** | **Expected prevalence, %**  **(95% CI)** |
| --- | --- |
| 10 – 29 | [3.8–31.9] |
| 30 – 49 | [4.3–22.6] |
| ≥ 50 | [6.6–29.5] |

**Age ≥ 75 years**

Overall, 135 centres included at least one patient aged 75 years or over. Among these, only 15 centres included 10 patients aged 75 or over. The following results are based on 205 patients aged 75 years or over admitted to the 15 centres.

**Table E7.** Prevalence of decisions of LST limitations in patients aged ≥ 75 years

| **Model** | **Proportion (95% CI)** |
| --- | --- |
| Fixed effect model | 0.4390 [0.3726-0.5077] |
| Random effects model | 0.4390 [0.3726-0.5077] |

Quantifying heterogeneity: tau^2 = 0; tau = 0; I^2 = 0.0% [0.0%; 53.6%]; H = 1.00 [1.00; 1.47]

Testing heterogeneity, p-value for the likelihood ratio test was 0.487.

**Figure E4.** Forest plot of prevalences of decisions of LST limitations in the 15 centres with ≥ 10 patients aged ≥ 75 years


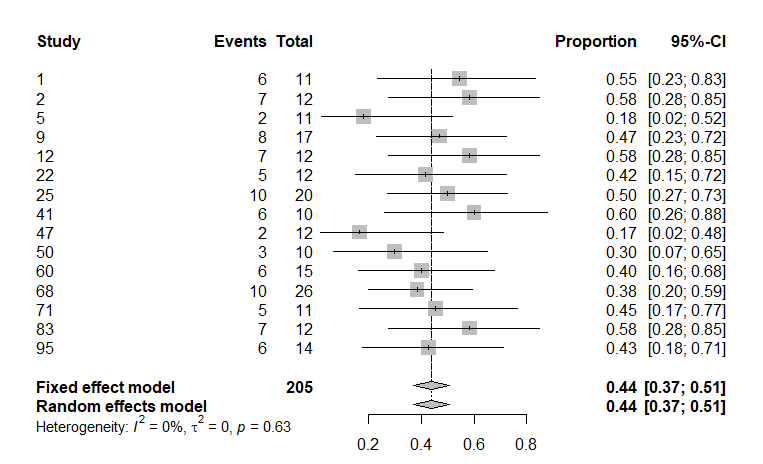


**References**

1. Le Gall JR, Lemeshow S, Saulnier F. A new Simplified Acute Physiology Score (SAPS II) based on a European/North American multicenter study. JAMA. 1993;270:2957–63.

2. Vincent JL, Moreno R, Takala J, et al. The SOFA (Sepsis-related Organ Failure Assessment) score to describe organ dysfunction/failure. On behalf of the Working Group on Sepsis-Related Problems of the European Society of Intensive Care Medicine. Intensive Care Med. 1996;22:707–10.

3. Juma S, Taabazuing M-M, Montero-Odasso M. Clinical frailty scale in an acute medicine unit: a simple tool that predicts length of stay. Can Geriatr J. 2016;19:34–9.

4. Vincent J-L, Rello J, Marshall J, et al. International study of the prevalence and outcomes of infection in intensive care units. JAMA. 2009;302:2323–9.

5. Taccone FS, Vangoethem N, Depauw R, et al. The role of organizational characteristics on the outcome of COVID-19 patients admitted to the ICU in Belgium. Lancet Regional Health - Europe. 2020;100019.
