# Supplementary material for: Limitation of life-sustaining therapies in critically ill patients with COVID-19: a descriptive epidemiological investigation from the COVID-ICU study
Source: Crit Care. 2023 Mar 11;27:103. doi: 10.1186/s13054-023-04349-1 (PMC10006561; doi:10.1186/s13054-023-04349-1)
Supplement: Supplementary file 2 — Additional file 2. Participating Sites and COVID–ICU Investigators. [file 13054_2023_4349_MOESM2_ESM.docx]

**Participating Sites and COVID–ICU Investigators**

CHU Angers, Angers, France (Alain Mercat, Pierre Asfar, François Beloncle, Julien Demiselle), APHP

- Hôpital Bicêtre, Le Kremlin-Bicêtre, France (Tài Pham, Arthur Pavot, Xavier Monnet, Christian

Richard), APHP - Hôpital Pitié Salpêtrière, Paris, France (Alexandre Demoule, Martin Dres, Julien

Mayaux, Alexandra Beurton), CHU Caen Normandie - Hôpital Côte de Nacre, Caen, France, (Cédric

Daubin, Richard Descamps, Aurélie Joret, Damien Du Cheyron), APHP - Hôpital Cochin, Paris, France

(Frédéric Pene, Jean-Daniel Chiche, Mathieu Jozwiak, Paul Jaubert), APHP - Hôpital Tenon, Paris

(France, Guillaume Voiriot, Muriel Fartoukh, Marion Teulier, Clarisse Blayau), CHRU de Brest – La

Cavale Blanche, Brest, France (Erwen L'Her, Cécile Aubron, Laetitia Bodenes, Nicolas Ferriere),

Centre Hospitalier de Cholet, Cholet, France (Johann Auchabie, Anthony Le Meur, Sylvain Pignal,

Thierry Mazzoni), CHU Dijon Bourgogne, Dijon, France (Jean-Pierre Quenot, Pascal Andreu, Jean-

Baptiste Roudau, Marie Labruyère), CHU Lille - Hôpital Roger Salengero, Lille, France (Saad Nseir,

Sébastien Preau, Julien Poissy, Daniel Mathieu), Groupe Hospitalier Nord Essonne, Longjumeau,

France (Sarah Benhamida, Rémi Paulet, Nicolas Roucaud, Martial Thyrault), APHM - Hopital Nord,

Marseille, France (Florence Daviet, Sami Hraiech, Gabriel Parzy, Aude Sylvestre), Hôpital de Melun-

Sénart, Melun, France (Sébastien Jochmans, Anne-Laure Bouilland, Mehran Monchi), Élément

Militaire de Réanimation du SSA, Mulhouse, France (Marc Danguy des Déserts, Quentin Mathais,

Gwendoline Rager, Pierre Pasquier), CHU Nantes - Hôpital Hotel Dieu, Nantes, France (Jean Reignier,

Amélie Seguin, Charlotte Garret, Emmanuel Canet), CHU Nice - Hôpital Archet, Nice, France (Jean

Dellamonica, Clément Saccheri, Romain Lombardi, Yanis Kouchit), Centre Hospitalier d'Orléans,

Orléans, France (Sophie Jacquier, Armelle Mathonnet, Mai-Ahn Nay, Isabelle Runge), Centre

Hospitalier Universitaire de la Guadeloupe, Pointe-à-Pitre, France (Frédéric Martino, Laure Flurin,

Amélie Rolle, Michel Carles), Hôpital de la Milétrie, Poitiers, France (Rémi Coudroy, Arnaud W

Thille, Jean-Pierre Frat, Maeva Rodriguez), Centre Hospitalier Roanne, Roanne, France (Pascal Beuret,

Audrey Tientcheu, Arthur Vincent, Florian Michelin), CHU Rouen - Hôpital Charles Nicolle, Rouen,

France (Fabienne Tamion, Dorothée Carpentier, Déborah Boyer, Gaetan Beduneau), CHRU Tours -

Hôpital Bretonneau, Tours, France (Valérie Gissot, Stéphan Ehrmann, Charlotte Salmon Gandonniere,

Djlali Elaroussi), Centre Hospitalier Bretagne Atlantique, Vannes, France (Agathe Delbove, Yannick

Fedun, Julien Huntzinger, Eddy Lebas), CHU Liège, Liège, Belgique (Grâce Kisoka, Céline Grégoire, Stella Marchetta, Bernard Lambermont), Hospices Civils de Lyon - Hôpital Edouard Herriot, Lyon,

France (Laurent Argaud, Thomas Baudry, Pierre-Jean Bertrand, Auguste Dargent), Centre Hospitalier

Du Mans, Le Mans, France (Christophe Guitton, Nicolas Chudeau, Mickaël Landais, Cédric Darreau),

Centre Hospitalier de Versailles, Le Chesnay, France (Alexis Ferre, Antoine Gros, Guillaume Lacave,

Fabrice Bruneel), Hôpital Foch, Suresnes, France (Mathilde Neuville, JérômeDevaquet, Guillaume

Tachon, Richard Gallot), Hôpital Claude Galien, Quincy sous Senart, France (Riad Chelha, Arnaud

Galbois, Anne Jallot, Ludivine Chalumeau Lemoine), GHR Mulhouse Sud-Alsace, Mulhouse, France

(Khaldoun Kuteifan, Valentin Pointurier, Louise-Marie Jandeaux, Joy Mootien), APHP - Hôpital

Antoine Béclère, Clamart, France (Charles Damoisel, Benjamin Sztrymf), APHP - Hôpital Pitié-

Salpêtrière, Paris, France (Matthieu Schmidt, Alain Combes, Juliette Chommeloux, Charles Edouard

Luyt), Hôpital Intercommunal de Créteil, Créteil, France (Frédérique Schortgen, Leon Rusel, Camille

Jung), Hospices Civils de Lyon - Hôpital Neurologique, Lyon, France (Florent Gobert), APHP - Hôpital

Necker, Paris, France (Damien Vimpere, Lionel Lamhaut), Centre Hospitalier Public du Cotentin -

Hôpital Pasteur, Cherbourg-en-cotentin, France (Bertrand Sauneuf, Liliane Charrrier, Julien Calus,

Isabelle Desmeules), CHU Rennes - Hôpital du Pontchaillou, Rennes, France (Benoît Painvin, Jean-

Marc Tadie), CHU Strasbourg - Hôpital Hautepierre, Strasbourg, France (Vincent Castelain, Baptiste

Michard, Jean-Etienne Herbrecht, Mathieu Baldacini), APHP - Hôpital Pitié Salpêtrière, Paris, France

(Nicolas Weiss, Sophie Demeret, Clémence Marois, Benjamin Rohaut), Centre Hospitalier Territorial

Gaston-Bourret, Nouméa, France (Pierre-Henri Moury, Anne-Charlotte Savida, Emmanuel Couadau,

Mathieu Série), Centre Hospitalier Compiègne-Noyon, Compiègne, France (Nica Alexandru), Groupe

Hospitalier Saint-Joseph, Paris, France (Cédric Bruel, Candice Fontaine, Sonia Garrigou, Juliette

Courtiade Mahler), Centre hospitalier mémorial de Saint-Lô, Saint-Lô, France (Maxime Leclerc,

Michel Ramakers), Grand Hôpital de l'Est Francilien, Jossigny, France (Pierre Garçon, Nicole Massou,

Ly Van Vong, Juliane Sen), Gustave Roussy, Villejuif, France (Nolwenn Lucas, Franck Chemouni,

Annabelle Stoclin), Centre Hospitalier Intercommunal Robert Ballanger, Aulnay-sous-Bois, France

(Alexandre Avenel, Henri Faure, Angélie Gentilhomme, Sylvie Ricome), Hospices Civiles de Lyon -

Hôpital Edouard Herriot, Lyon, France (Paul Abraham, Céline Monard, Julien Textoris, Thomas

Rimmele), Centre Hospitalier d'Avignon, Avignon, France (Florent Montini), Groupe Hospitalier

Diaconesses - Croix Saint Simon, Paris, France (Gabriel Lejour, Thierry Lazard, Isabelle Etienney,

Younes Kerroumi), CHU Clermont-Ferrand - Hôpital Gabriel Montpied, Clermont Ferrand, France (Claire Dupuis, Marine Bereiziat , Elisabeth Coupez, François Thouy), Hôpital d'Instruction des

Armées Percy, Clamart, France (Clément Hoffmann, Nicolas Donat, Anne Chrisment, Rose-Marie

Blot), CHU Nancy - Hôpital Brabois, Vandoeuvre-les-Nancy, France (Antoine Kimmoun, Audrey

Jacquot, Matthieu Mattei, Bruno Levy), Centre Hospitalier de Vichy, Vichy, France (Ramin Ravan,

Loïc Dopeux, Jean-Mathias Liteaudon, Delphine Roux), Hopital Pierre Bérégovoy, Nevers, France

(Brice Rey, Radu Anghel, Deborah Schenesse , Vincent Gevrey), Centre Hospitalier de Tarbes, Tarbes,

France (Jermy Castanera, Philippe Petua, Benjamin Madeux), Hôpitaux Civils de Colmar - Hôpital

Louis pasteur, Colmar, France (Otto Hartman), CHU Charleroi - Hôpital Marie Curie, Bruxelles,

Belgique (Michael Piagnerelli, Anne Joosten,Cinderella Noel, Patrick Biston), Centre hospitalier de

Verdun Saint Mihiel, Saint Mihiel, France (Thibaut Noel), CH Eure-Seine - Hôpital d'Evreux-Vernon,

Evreux, France (Gurvan LE Bouar, Messabi Boukhanza, Elsa Demarest, Marie-France Bajolet), Hôpital

René Dubos, Pontoise, France (Nathanaël Charrier, Audrey Quenet, Cécile Zylberfajn, Nicolas Dufour),

APHP - Hôpital Lariboisière, Paris, France (Buno Mégarbane, Sébastian Voicu, Nicolas Deye, Isabelle

Malissin), Centre Hospitalier de Saint-Brieuc, Saint-Brieuc, France (François Legay, Matthieu Debarre,

Nicolas Barbarot, Pierre Fillatre), Polyclinique Bordeaux Nord Aquitaine, Bordeaux, France (Bertrand

Delord, Thomas Laterrade, Tahar Saghi, Wilfried Pujol), HIA Sainte Anne, Toulon, France (Pierre

Julien Cungi, Pierre Esnault, Mickael Cardinale), Grand Hôpital de l'Est Francilien, Meaux, France

(Vivien Hong Tuan Ha, Grégory Fleury, Marie-Ange Brou, Daniel Zafimahazo), HIA Robert Picqué,

Villenave d'Ornon, France (David Tran-Van, Patrick Avargues, Lisa Carenco), Centre Hospitalier

Fontainebleau, Fontainebleau, France (Nicolas Robin, Alexandre Ouali, Lucie Houdou), Hôpital

Universitaire de Genève, Genève, Suisse (Christophe Le Terrier, Noémie Suh, Steve Primmaz, Jérome

Pugin), APHP - Hôpital Beaujon, Clichy, France (Emmanuel Weiss, Tobias Gauss, Jean-Denis Moyer,

Catherine Paugam Burtz), Groupe Hospitalier Bretage Sud, Lorient, France (Béatrice La Combe,

Rolland Smonig, Jade Violleau, Pauline Cailliez), Centre Hospitalier Intercommunal Toulon, La Seyne

sur Mer, France (Jonathan Chelly), Centre Hospitalier de Dieppe, Dieppe, France (Antoine

Marchalot, Cécile Saladin, Christelle Bigot), CHU de Martinique, Fort-de-France, France (Pierre-

Marie Fayolle, Jules Fatséas, Amr Ibrahim, Dabor Resiere), Hôpital Fondation Adolphe de Rothchild,

Paris, France (Rabih Hage, Clémentine Cholet, Marie Cantier, Pierre Trouiler), APHP - Bichat Claude

Bernard, Paris, France (Philippe Montravers, Brice Lortat-Jacob, Sebastien Tanaka, Alexy Tran Dinh),

APHP - Hôpital Universitaire Paris Sud, Bicêtre, France (Jacques Duranteau, Anatole Harrois, Guillaume Dubreuil, Marie Werner), APHP - Hôpital Européen Georges Pompidou, Paris, France

(Anne Godier, Sophie Hamada, Diane Zlotnik, Hélène Nougue) , APHP, GHU Henri Mondor, Créteil,

France (Armand Mekontso-Dessap, Guillaume Carteaux, Keyvan Razazi, Nicolas De Prost), APHP -

Hôpitaux Universitaires Henri Mondor, Créteil, France (Nicolas Mongardon, Meriam Lamraoui, Claire

Alessandri, Quentin de Roux), APHP - Hôpital Lariboisière, Paris, France (Charles de Roquetaillade,

Benjamin G. Chousterman, Alexandre Mebazaa, Etienne Gayat), APHP - Hôpital Saint-Antoine, Paris,

France (Marc Garnier, Emmanuel Pardo, LeaSatre-Buisson, Christophe Gutton), APHP Hôpital Saint-

Louis, Paris, France (Elise Yvin, Clémence Marcault, Elie Azoulay, Michael Darmon), APHP - Hôpital

Saint-Antoine, Paris, France (Hafid Ait Oufella, Geoffroy Hariri, Tomas Urbina, Sandie Mazerand),

APHP - Hôpital Raymond Pointcarré, Garches, France (Nicholas Heming, Francesca Santi, Pierre

Moine, Djillali Annane), APHP - Hôpital Pitié Salpêtrière, Paris, France (Adrien Bouglé, Edris Omar,

Aymeric Lancelot, Emmanuelle Begot), Centre Hospitalier Victor Dupouy, Argenteuil, France (Gaétan

Plantefeve, Damien Contou, Hervé Mentec, Olivier Pajot), CHU Toulouse - Hôpital Rangueil,

Toulouse, France (Stanislas Faguer, Olivier Cointault, Laurence Lavayssiere, Marie-Béatrice Nogier),

Centre Hospitalier de Poissy, Poissy, France (Matthieu Jamme, Claire Pichereau, Jan Hayon, Hervé

Outin), APHP - Hôpital Saint-Louis, Paris, France (François Dépret, Maxime Coutrot, Maité

Chaussard, Lucie Guillemet), Clinique du MontLégia, CHC Groupe-Santé, Liège, Belgique (Pierre

Goffin, Romain Thouny, Julien Guntz, Laurent Jadot), CHU Saint-Denis, La Réunion, France (Romain

Persichini), Centre Hospitalier de Tourcoing, Tourcoing, France (Vanessa Jean-Michel, Hugues

Georges, Thomas Caulier), Centre Hospitalier Henri Mondor d'Aurillac, Aurillac, France (Gaël Pradel,

Marie-Hélène Hausermann, Thi My Hue Nguyen-Valat, Michel Boudinaud), Centre Hospitalier Saint

Joseph Saint Luc, Lyon, France (Emmanuel Vivier, SylvèneRosseli, Gaël Bourdin, Christian Pommier)

Centre Hospitalier de Polynésie Française, Polynésie, France (Marc Vinclair, Simon Poignant, Sandrine

Mons), Ramsay Générale de Santé, Hôpital Privé Jacques Cartier, Massy, France (Wulfran Bougouin),

Centre Hospitalier Alpes Léman, Contamine sur Arve, France (Franklin Bruna, Quentin Maestraggi,

Christian Roth), Hospices Civils de Lyon - Hôpital de la Croix Rousse, Lyon, France (Laurent Bitker,

François Dhelft, Justine Bonnet-Chateau, Mathilde Filippelli), Centre Cardiologique du Nord, Saint-

Denis, France (Tristan Morichau-Beauchant, Stéphane Thierry, Charlotte Le Roy, Mélanie Saint

Jouan), GHU - Hôpital Saint-Anne, Paris, France (Bruno Goncalves, Aurélien Mazeraud, Matthieu

Daniel, Tarek Sharshar) CHR Metz - Hôpital Mercy, Metz, France (Cyril Cadoz, Rostane Gaci, Sébastien Gette, Guillaune Louis), APHP - Hôpital Paul Brousse, Villejuif, France (Sophe-Caroline

Sacleux, Marie-Amélie Ordan), CHRU Nancy - Hôpital Central, Nancy, France (Aurélie Cravoisy,

Marie Conrad, Guilhem Courte, Sébastien Gibot), Centre Hospitalier d’Ajaccio, Ajaccio, France

(Younès Benzidi, Claudia Casella, Laurent Serpin, Jean-Lou Setti), Centre Hospitalier de Bourges,

Bourges, France (Marie-Catherine Besse, Anna Bourreau), Centre hospitalier de la Côte Basque,

Bayonne, France (Jérôme Pillot, Caroline Rivera, Camille Vinclair, Marie-Aline Robaux), Hospices

Civils de Lyon - Hôpital de la Croix Rousse, Lyon, France (Chloé Achino, Marie-Charlotte Delignette,

Tessa Mazard, Frédéric Aubrun), CH Saint-Malo, Saint-Malo, France (Bruno Bouchet, Aurélien Frérou,

Laura Muller, Charlotte Quentin), Centre Hospitalier de Mulhouse, Mulhouse, France (Samuel Degoul),

Centre Hospitalier de Briançon, Briançon, France (Xavier Stihle, Claude Sumian, Nicoletta Bergero,

Bernard Lanaspre), CHU Nice, Hôpital Pasteur 2, Nice, France (Hervé Quintard, Eve Marie Maiziere),

Centre Hospitalier des Pays de Morlaix, Morlaix, France (Pierre-Yves Egreteau, Guillaume Leloup,

Florin Berteau, Marjolaine Cottrel), Centre Hospitalier Valence, Valence, France (Marie Bouteloup,

Matthieu Jeannot, Quentin Blanc, Julien Saison), Centre Hospitalier Niort, Niort, France (Isabelle

Geneau, Romaric Grenot, Abdel Ouchike, Pascal Hazera), APHP - Hôpital Pitié Salpêtrière, Paris,

France (Anne-Lyse Masse, Suela Demiri, Corinne Vezinet, Elodie Baron, Deborah Benchetrit, Antoine

Monsel), Clinique du Val d'Or, Saint Cloud, France (Grégoire Trebbia, Emmanuelle Schaack, Raphaël

Lepecq, Mathieu Bobet), Centre Hospitalier de Béthune, Béthune, France (Christophe Vinsonneau,

Thibault Dekeyser, Quentin Delforge, Imen Rahmani), Groupe Hospitalier Intercommunal de la Haute-

Saône, Vesoul, France (Bérengère Vivet, Jonathan Paillot, Lucie Hierle, Claire Chaignat, Sarah

Valette), Clinique Saint-Martin, Caen, France (Benoït Her, Jennifier Brunet), Ramsay Générale de

Santé, Clinique Convert, Bourg en Bresse, France (Mathieu Page, Fabienne Boiste, Anthony Collin),

Hôpital Victor Jousselin, Dreux, France(Florent Bavozet, Aude Garin ,Mohamed Dlala , Kais Mhamdi),

Centre Hospitalier de Troye, Troye, France, (Bassem Beilouny, Alexandra Lavalard, Severine Perez),

CHU de ROUEN-Hôpital Charles Nicolle, Rouen, France (Benoit Veber, Pierre-Gildas Guitard,

Philippe Gouin, Anna Lamacz), Centre Hospitalier Agen-Nérac, Agen, France (Fabienne Plouvier,

Bertrand P Delaborde, Aïssa Kherchache, Amina Chaalal), APHP - Hôpital Louis Mourier, Colombes,

France (Jean-Damien Ricard, Marc Amouretti, Santiago Freita-Ramos, Damien Roux), APHP - Hôpital

Pitié-Salpêtrière, Paris, France (Jean-Michel Constantin, Mona Assefi, Marine Lecore, Agathe Selves),

Institut Mutualiste Montsouris, Paris, France (Florian Prevost, Christian Lamer, Ruiying Shi, Lyes Knani), CHU Besançon – Hôpital Jean Minjoz, Besançon, France, (Sébastien Pili Floury, Lucie

Vettoretti), APHP - Hôpital Universitaire Robert-Debré, Paris, France (Michael Levy, Lucile Marsac,

Stéphane Dauger, Sophie Guilmin-Crépon), CHU Besançon – Hôpital Jean Minjoz, Besançon, France,

(Hadrien Winiszewski, Gael Piton, Thibaud Soumagne, Gilles Capellier) ; Médipôle Lyon-

Villeurbanne, Vileurbanne, France, (Jean-Baptiste Putegnat, Frédérique Bayle, Maya Perrou, Ghyslaine

Thao), APHP - Ambroise Paré, Boulogne-Billancourt, France (Guillaume Géri, Cyril Charron, Xavier

Repessé, Antoine Vieillard-Baron), CHU Amiens Picardie, Amiens, France (Mathieu Guilbart, Pierre-

Alexandre Roger, Sébastien Hinard, Pierre-Yves Macq), Hôpital Nord-Ouest, Villefranche-sur-Saône,

France (Kevin Chaulier, Sylvie Goutte), CH de Châlons en Champagne, Châlons en Champagne,

France (Patrick Chillet, Anaïs Pitta, Barbara Darjent, Amandine Bruneau), CHU Angers, Angers,

France (Sigismond Lasocki, Maxime Leger, Soizic Gergaud, Pierre Lemarie), CHU Grenoble Alpes,

Grenoble, France (Nicolas Terzi, Carole Schwebel, Anaïs Dartevel, Louis-Marie Galerneau), APHP -

Hôpital Européen Georges Pompidou, Paris, France (Jean-Luc Diehl, Caroline Hauw-Berlemont,

Nicolas Péron, Emmanuel Guérot), Hôpital Privé d'Antony, Antony, France (Abolfazl Mohebbi Amoli,

Michel Benhamou, Jean-Pierre Deyme, Olivier Andremont), Institut Arnault Tzanck,Saint Laurent du

Var, France (Diane Lena, Julien Cady, Arnaud Causeret, Arnaud De La Chapelle) ; Centre Hospitalier

d’ Angoulême, Angoulême, France (Christophe Cracco, Stéphane Rouleau, David Schnell) ; Centre

Hospitalier de Cahors, Cahors, France (Camille Foucault), Centre hospitalier de Carcassonne,

Carcassonne, France (Cécile Lory) ; CHU Nice – Hôpital L’Archet 2, Nice, France (Thibault Chapelle,

Vincent Bruckert, Julie Garcia, Abdlazize Sahraoui) ; Hôpital Privé du Vert Galant, Tremblay-en-

France, France (Nathalie Abbosh, Caroline Bornstain, Pierre Pernet) ; Centre Hospitalier de

Rambouillet, Rambouillet, France (Florent Poirson, Ahmed Pasem, Philippe Karoubi) ; Hopitaux du

Léman, Thonon les Bains, France (Virginie Poupinel, Caroline Gauthier, François Bouniol, Philippe

Feuchere), Centre Hospitalier Victor Jousselin, Dreux, France (Florent Bavozet, Anne Heron), Hôpital

Sainte Camille, Brie sur Marne, France (Serge Carreira, Malo Emery, Anne Sophie Le Floch, Luana

Giovannangeli), Hôpital d’instruction des armées Clermont-Tonnerre, Brest, France (Nicolas Herzog,

Christophe Giacardi, Thibaut Baudic, Chloé Thill), APHP - Hôpital Pitié Salpêtrière, Paris, France

(Said Lebbah, Jessica Palmyre, Florence Tubach, David Hajage) ; APHP - Hôpital Avicenne, Bobigny,

France (Nicolas Bonnet, Nathan Ebstein, Stéphane Gaudry, Yves Cohen) ; Groupement Hospitalier la

Rochelle Ré Amis, La Rochelle, France (Julie Noublanche, Olivier Lesieur) ; Centre Hospitalier Intercommunal de Mont de Marsan et du Pays des Sources, Mont de Marsan, France (Arnaud Sément,

Isabel Roca-Cerezo, Michel Pascal, Nesrine Sma) ; Centre Hospitalier Départemental de Vendée, La-

Roche-Sur-Yon, France (Gwenhaël Colin, Jean-Claude Lacherade, Gauthier Bionz, Natacha

Maquigneau) ; Pôle Anesthésie-Réanimation, CHU Grenoble (Pierre Bouzat, Michel Durand, Marie-

Christine Hérault, Jean-Francois Payen)
